# Homogenization method for microscopic characterization of the composite magnetoelectric multiferroics

**DOI:** 10.1038/s41598-020-57977-w

**Published:** 2020-01-28

**Authors:** K. P. Jayachandran, J. M. Guedes, H. C. Rodrigues

**Affiliations:** IDMEC, Iinstituto Superior Técnico, Universidade de Lisboa, Av. Rovisco Pais, 1049-001 Lisboa, Portugal

**Keywords:** Energy science and technology, Energy harvesting, Engineering, Mechanical engineering, Materials science, Ferroelectrics and multiferroics, Mathematics and computing, Computational science

## Abstract

Tuning of magnetization or electrical polarization using external fields other than their corresponding conjugate fields (i.e., magnetic field for the former or electric field for the latter response) attracts renewed interest due to its potential for applications. The magnetoelectric effect in multiferroic 1–3 composite composed of alternating magnetic and ferroelectric layers operating in linear regime consequent to external biasing fields is simulated and analysed theoretically. Two-scale homogenization procedure to arrive at the equilibrium overall physical properties of magnetoelectric multiferroic composite is formulated using variational analysis. This procedure is extended to quantify the underlying *local* (microscopic) electric, magnetic and elastic fields and thereby compute local distribution of stresses and strains, electrical and magnetic potentials, the electric and magnetic fields as well as the equivalent von Mises stresses. The computational model is implemented by modifying the software POSTMAT (*material postprocessing*). Computed local stress/strain profiles and the von Mises stresses consequent to biasing electrical and magnetic fields provide insightful information related to the magnetostriction and the ensuing electrical and magnetic polarization. Average polarization and magnetization against magnetic and electric fields respectively are computed and found to be in reasonable limits of the experimental results on similar composite systems. The homogenization model covers multiferroics and its composites regardless of crystallographic symmetry (with the caveat of assuming an ideal and semi-coherent interface connecting the constituent phases) and offer computational efficiency besides unveiling the nature of the underlying microscopic field characteristics.

## Introduction

Multiferroics are of great scientific and technological interest due to their magnetoelectric properties originating from the coupling between its ferroelectric (FE) and ferromagnetic (FM) order parameters^1–4^. Magnetoelectric effect, proposed by Landau and Lifshitz in the late 1950s^5^, manifests in a linear relation between magnetic and electric fields in matter and it causes magnetic induction of polarization **P** or electric induction of magnetization **M**. Thereafter Dzyaloshinskii^6^ by symmetry considerations predicted this effect and subsequently observed experimentally by Astrov^7^. Besides the existence of cross-coupling of fields, there exists systems in which two types of ordering-ferromagnetism, the spontaneous ordering of orbital and spin magnetic moments, and ferroelectricity, the spontaneous ordering of electric dipole moments- coexist in one material in the absence of external electric and magnetic fields^8^. They show promising applications in the field of magnetoelectric sensors, four-sate logic memories, etc., owing to its possession of coupled electrical and magnetic degrees of freedom^9–11^. Yet, the available single phase multiferroics impair from weak magnetoelectric coupling and lower (than ambient) transition temperatures^12^.

The most obvious strategy to engineer multiferroics with reasonable coupling and transition temperatures (both magnetic and ferroelectric) involves combining both magnetic and FE materials into a composite^13^. A non-magnetic FE such as BaTiO_3_ (BTO) is combined with a non-FE magnetic material such as CoFe_2_O_4_ (CFO) to exhibit a “product property” called magnetoelectric effect which neither of the phases possess individually^14^. Spinel magnetic oxides such as CFO possesses a magnetic transition temperature (*T*_*N*_) much higher than room temperature, have good coherence with FE oxides such as BTO in the crystal structure which guarantees preparation of high-quality FE–FM films.

Most prominent aspect of the multiferroics is their magnetoelectric coupling. A magnetic field induces a change in the shape of the ferromagnetic phase (a property called *magnetostriction*), which in turn stresses the piezoelectric phase in which an electric polarization **P** is generated. This magnetoelectric effect is characterised as *direct magnetoelectric effect*. *Converse magnetoelectric effect* is customarily the generation of magnetization **M** due to an applied electric field. In both cases the coupling is mechanical in nature although various other forms such as electrical, chemical, electronic etc., are possible too^15^. For instance, electric field can induce appreciable variations in magnetic properties of thinfilm ferromagnetic systems as shown in^16^. While a ferroic property (e.g., electrical or magnetic polarization) is modified using its conjugate fields (electric or magnetic fields respectively), in a magnetoelectric material magnetic field can alter electric polarization and an electric field can alter magnetization. We investigate this aspect of the magnetoelectric composites in more detail in this article since the tuning of polarization or magnetization using fields other than the respective conjugate fields offer potential for numerous applications in bulk and thinfilm forms^15,17^.

Several methods have been attempted to achieve strain-induced magnetoelectric coupling in FE–FM composites^12,18^. Vertically aligned nanocomposites where spinel magnetic oxide nanopillars embedded epitaxially in a FE phase in which the coupling is mediated through epitaxial strains is explored to attain strong elastic coupling^19^. It has generally been accepted that a magnetic field is the only means to switch magnetization and to sustain this property in magnetic materials. However, the energy and space consumption entail the usage of magnets for this purpose is prohibitive in the context of demands of miniaturization and energy economy^15^. Nonmagnetic routes such as through chemical doping, strain, current, light as well as electric field are found to be robust, compatible, economic, reversible and nonvolatile. A number of studies (mostly experimental and few theoretical) on the modulation and switching of magnetism have emerged in recent years. A compilation of the experimental works on voltage control of magnetism focussing on thinfilm/thick film heterostructures can be seen in e.g., Vaz^12^, Song *et al*.^15^, Guo *et al*.^20^, and Taniyama^21^, etc. When a ferroelectric material replaces the nonmagnetic layer, additional strain transfer can be induced by an electric field due to the inverse piezoelectric effect with a possible polarization switching^21^. This additional strain transfer reinforces the sway of magnetic properties by an electrical field. Moreover, the hysteretic electric field-polarization profile of the ferroelectric material would sustain the magnetization in contrast to the FM/dielectric interface where the magnetization disappears once the electric field is switched off. This property would be beneficial for the non-volatile memory applications as is perceived recently by Taniyama^21^. Zavaliche *et al*., through piezoelectric force and magnetic force microscopy imaging observed room temperature reversal of magnetization by electric field in epitaxial ferroelectric- ferrimagnetic nanostructures^22^.

One among the earliest works on electrical manipulation of magnetization was that of Chiba *et al*., where the magnetic anisotropy which determines the **M** direction is controlled by applying an electric field on a metal–insulator–semiconductor structure^23^. Chopdekar and Suzuki have synthesised epitaxial CFO layer on single crystal BTO substrate in a model heterostructure for magnetoelectric composite^24^. Sahoo *et al*. show that the magnetic properties of a transition metal ferromagnetic thin film can be strongly altered with a single crystal ferroelectric BTO^25^. Magnetoelectric interactions has been investigated under a dc electric field in Ni-lead zirconante titanate (PZT) bilayer by Fetisov *et al*. and found a 40% increase in ME voltage^26^. Hu *et al*., demonstrate a full reversal of perpendicular magnetization via successive precession and damping, driven by a perpendicular electric-field through a phase-field simulation in a multiferroic nanostructure^27^.

The magnetoelectric coupling can occur in response to an applied magnetic field as well by the confluence of magnetostrictive effects and piezoelectricity to achieve an electrical response. The magnetic field induced electric polarization is the main working mechanism of magnetic field sensing^4^. Nan *et al*., have shown that the magnetic field induced polarization in bulk composites is lower than that in thin film nanostructures using Green’s function approach^28^. Srinivasan *et al*., have shown agreement of magnetoelectric property dependence on volume fraction and bias magnetic field through theoretical analysis of bilayers and by synthesized thick films of PZT–nickel ferrite (NFO) prepared by tape casting^29^. Burton and Tsymbal demonstrate a different type of interface magnetoelectric effect at the interface between a metallic-doped La manganite and BTO using first-principles calculations^30^. Pertsev described theoretically using a nonlinear thermodynamic approach, a strain-driven spin reorientation transition may be induced in epitaxial ferromagnetic films by a moderate electric field applied to a ferroelectric substrate^31^.

The magnetoelectric effect in multiferroic composites dependent on their microstructure and coupling interaction across the ferroelectric- magnetic interface^28^. Landau’s phenomenological theory show that magnetization is coupled with electric field via inverse magnetoelectric coupling constant *α* and it allows room for modulation of magnetization by electric field^21^. Uetsuji *et al*. have demonstrated the macroscopic magnetoelectric effect due to mechanical strain between the FE and ferromagnetic phases in an inhomogeneous microstructure through multiscale finite element simulations^32^. Further survey on important works done on this materials is given in Supplementary Information. The local or microscopic fields are composed of external applied fields (which are designated as “average fields”) and local variables and are related by governing equations.

In the present model, one phase (fibre) of the composite is polycrystalline CFO which obviously is an aggregate of grains and should be textured to deliver its magnetic polarization. The BTO (i.e., matrix) is single crystalline and the electric polarization is oriented out-of-plane (along the *y*_3_-axis) of the composite layer and so is the magnetization of CFO. Nonetheless, the interface these phases share is complex in that sense, and would not fit into the conditions for a coherent interface where the two crystalline phases should possess a close crystallographic registry. Moreover thanks to the large misfit (≈0.52),^33^ due to stark contrast in lattice parameters of BTO and CFO, it becomes energetically favourable to form a semi-coherent interface where the mismatch is periodically reconciled by misfit dislocations^34^. In nanocrystal aggregates, however, since a large fraction of atoms exist in the interface regions, the thermodynamic stability of coherent interfaces with close crystallographic registry dominates the overall stability of the system^35^. Theoretical work on less-than ideal interface coupling suggests that in-plane magnetoelectric interface is limiting the magnitude of the ME coupling and attribute this to the clamping effect of the substrate on the ferroelectric phase and to the interface quality quantified by a coupling parameter *k*^36^. Since the amount of strain that can be imparted by the ferroelectric phase is limited via this in-plane interfacial geometry. However, thinfilm fibre reinforced nanostructures, in which the interface is perpendicular to the substrate, remove the effect of substrate clamping and allow for better strain induced coupling between the two phases^19^. The present system since not being thinfilm on a substrate thus naturally is immune to substrate clamping albeit susceptible to the effect of interface. Interactions across the interface can become quite complex with changes in local chemical environment, bond angle and length, electron density, orbital order, etc. Studies on individual magnetic and ferroelectric films demonstrate that there exist a critical thickness of 6 unit cells (of the order of ≈24 Å) and 13 unit cells respectively, above which system exhibits bulk-like properties^37,38^. A tangible point derived from this discussion is that a comprehensive treatment of the interface effects in magnetoelectric composites warrants an atomistically enriched model possessing more resolution which scales down to crystallographic cell size (of few Angstroms). Since the applications pertaining to the magnetoelectrics mostly involve electrical/magnetic loading, the issues during mechanical load transfer i.e., either the shear failure (when the interfacial bonding is weak) or tensile failure (due to strong interfacial bonding) is not prevalent here. The forces of interfacial bonding are van der Waals forces which quickly vanish at longer distances between interacting molecules (e.g., ref. ^39^) and thus will diminish at the realm of thickness that the magnetoelectric composite in the present model operates. The impact of interface is critical for composite configurations where the thicknesses of the individual phases are less than a critical thickness. With greater thicknesses of the phases as in the present case where the constituents are of the order of micrometers which exceeds the critical thickness, goes to relaxed growth where the interfacial misfit strain energy (due to elastic mismatch) is marginal and the ensuing stress is released.

**Figure 1:**
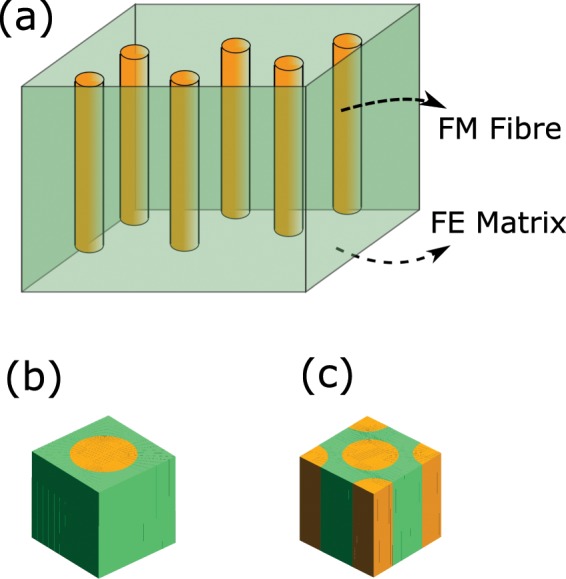
(**a**) Schematic diagram of a 1–3 magnetoelectric composite where ferromagnetic (FM) fibres are embedded in a ferroelectric (FE) matrix. Two microstructures (unit cells) (**b**) a customary fibre-reinforced matrix and (**c**) a honeycomb lattice carved out of the 1–3 composite as representative volume element. Images drawn using Inkscape 0.92.4 (https://www.inkscape.org) under GNU General Public License and Gmsh version 2.15.0 (http://gmsh.info/) under GNU General Public License.

The stress/strain field associated with electrostrain/magnetostrictive strain change the magnetization/polarization of the composite. Strain engineering is an ideal method for controlling and modulating magnetism. The magnetoelectric coupling is plausibly through strain transfer from FE layer to ferromagnetic layer which will induce (or enhance) magnetic properties in this system. Due to the inverse piezoelectric effect, the electric field applied to the FE material strain it and this strains gets migrated to the FM phase and this will eventually produce a magnetization due to piezomagnetism. In this paper we analyse the distribution of local fields in a fibre-reinforced magnetoelectric composite of 1–3 connectivity due to the application of external electric and magnetic fields. A schematic diagram depicting the FM fibres embedded in a matrix of FE material is shown in Fig. 1. A homogenization method is developed for multiferroics of lowest crystallographic symmetry through a two-scale asymptotic analysis of the microscopic electric, magnetic and mechanical fields to profiling the impact on each of them of an external field in a microscopic scale. This method is applied to a magnetoelectric composite assumed to be possessing an ideal and semi-coherent interface connecting the constituent FE and FM phases. In order to denote the robustness of the model algorithm, we used two microstructures representing the 1–3 configuration *viz*., the customary fibre-reinforced composite architecture and the honeycomb lattice as depicted in Figs. 1(b) and (c) respectively. The unit cells are so chosen that they can encompass the heterogeneity of the composite comprehensively.

## Theory

### Microscopic and average fields

Let Ω be a fixed domain in **x**–space. We consider an auxiliary **y**–space divided into parallelepiped periods **Y**. For a linear anisotropic magnetoelectric material, generated through the periodic repetition of a *unit cell* representing the smallest sample of heterogeneity of the material domain Ω, the constitutive equations of the multiferroic magnetoelectric material and the asymptotic homogenization method can be found in the Supplementary Information. The microscopic fields (or local fields) pertaining to the electrical, magnetic, and spatial degrees of freedom and their coupling interactions at an arbitrary point in the unit cell can be obtained by the asymptotic analysis^40^. Let *ε* be the asymptotic scale factor representing the microstructure scale such that **y** = **x**/*ε*. the displacement **u**, electric potential *φ* and magnetic potential *ψ* are expanded asymptotically up to the first-order variation terms, giving

1 $$\left.{\begin{array}{ccc}{{\bf{u}}}^{\varepsilon }({\bf{x}}) & = & {{\bf{u}}}^{0}({\bf{x}},{\bf{y}})+\varepsilon {{\bf{u}}}^{1}({\bf{x}},{\bf{y}})\\ {\phi }^{\varepsilon }({\bf{x}}) & = & {\phi }^{0}({\bf{x}},{\bf{y}})+\varepsilon {\phi }^{1}({\bf{x}},{\bf{y}})\\ {\psi }^{\varepsilon }({\bf{x}}) & = & {\psi }^{0}({\bf{x}},{\bf{y}})+\varepsilon {\psi }^{1}({\bf{x}},{\bf{y}})\end{array}}\right\},{\bf{y}}={\bf{x}}/\varepsilon ,$$

where **u**^1^(**x**, **y**), *φ*^1^(**x**, **y**) and *ψ*^1^(**x**, **y**) are functions to be determined which are **Y**- periodic in **y**.

The microscopic fields at an arbitrary point of the unit cell can be determined using the Supplementary equations Eqs. S11 and S6. The displacement field **u**^*ε*^(**x**), the electric potential field *φ*^*ε*^(**x**) and the magnetic potential field *ψ*^*ε*^(**x**) involving details of the microstructure are given by

2 $$\begin{array}{lll}{{\bf{u}}}^{\varepsilon }({\bf{x}}) & = & {{\bf{u}}}^{0}({\bf{x}})+\varepsilon \left[{\chi }_{k}^{mn}({\bf{x}},{\bf{y}}) \right.\\  &  & \times \,{\epsilon }_{mn}({u}^{0}({\bf{x}}))+{\Phi }_{k}^{m}({\bf{x}},{\bf{y}})\frac{{\rm{\partial }}{\phi }^{0}({\bf{x}})}{{\rm{\partial }}{x}_{m}}\\  &  & \left.+\,{\Gamma }_{k}^{m}({\bf{x}},{\bf{y}})\frac{{\rm{\partial }}{\psi }^{0}({\bf{x}})}{{\rm{\partial }}{x}_{m}} \right] \end{array}$$

3 $$\begin{array}{ccc}{\phi }^{\varepsilon }({\bf{x}}) & = & {\phi }^{0}({\bf{x}})+\varepsilon \left[{\eta }^{mn}({\bf{x}},{\bf{y}}) \right. \\  &  & \times \,{\epsilon }_{mn}({u}^{0}({\bf{x}}))+{R}^{m}({\bf{x}},{\bf{y}})\frac{{\rm{\partial }}{\phi }^{0}({\bf{x}})}{{\rm{\partial }}{x}_{m}}\\  &  & \left. +\,{\Psi }^{m}({\bf{x}},{\bf{y}})\frac{{\rm{\partial }}{\psi }^{0}({\bf{x}})}{{\rm{\partial }}{x}_{m}}\right]\end{array}$$

4 $$\begin{array}{ccc}{\psi }^{\varepsilon }({\bf{x}}) & = & {\psi }^{0}({\bf{x}})+\varepsilon \left[{\lambda }^{mn}({\bf{x}},{\bf{y}}) \right. \\  &  & \times \,{\epsilon }_{mn}({u}^{0}({\bf{x}}))+{\Theta }^{m}({\bf{x}},{\bf{y}})\frac{{\rm{\partial }}{\phi }^{0}({\bf{x}})}{{\rm{\partial }}{x}_{m}}\\  &  & \left. +\,{Q}^{m}({\bf{x}},{\bf{y}})\frac{{\rm{\partial }}{\psi }^{0}({\bf{x}})}{{\rm{\partial }}{x}_{m}}\right]\end{array}$$

Here **χ**, *R* and *Q* are microscopic characteristic material, electric and magnetic displacements respectively. Φ, Γ, *η*, *Ψ*, *λ* and Θ are characteristic coupled functions. It is postulated that **u**^0^ is constant with respect to **y**, and depends only on **x** and hence can be equally valid for other fields *φ*^0^ and *ψ*^0^^41^. The local strain ${\epsilon }_{ij}^{\varepsilon }({\bf{x}})$, electric field ${E}_{j}^{\varepsilon }({\bf{x}})$ and the magnetic field ${H}_{j}^{\varepsilon }({\bf{x}})$ too can be obtained in a similar way once the homogenized macroscopic problem is solved (and are given in Supplementary Information). For the complete description of the state of stress, or for that matter, state of the electrical and magnetic field impacts within a body we require to know the stress and other fields at every point of the body. The microscopic stress ${\sigma }_{ij}^{\varepsilon }({\bf{x}})$, electrical displacement ${D}_{i}^{\varepsilon }({\bf{x}})$ and magnetic flux densities ${B}_{i}^{\varepsilon }({\bf{x}})$ at each point of the domain can be computed and is given in Supplementary Information. The asymptotic expansion in Eq. 1 means that the fields **u**^*ε*^, *φ*^*ε*^ and *ψ*^*ε*^ are the smooth functions plus the trailing little perturbing terms.

The equivalence of the average stress and homogenised stress can easily be expressed by applying the average operator 〈⋅〉 denoting $(\frac{1}{\left|Y\right|}{\int }_{Y}.dY)$, where *Y* is the domain size. Hence the average fields 〈*σ*_*ij*_〉, 〈*D*_*i*_〉 and 〈*B*_*i*_〉 are computed and given in Supplementary Information.

## Numerical Results and Discussion

The methodology used to obtain the average and local fields is based on the software POSTMAT(*material postprocessing*) developed by Guedes and Kikuchi^40^. It is to be noticed that the homogenized coefficients only depend on the local (microscopic) structure of the medium, and is obtained by the numerical solution of the boundary value problem, where the boundary conditions being of the periodic type. The homogenization method gives relevant information on the local and global behaviours in contrast to majority of problems in mechanics where the micro- and macro-processes are of very different nature^41^. Microscopic cell problems are solved prior to the macroscopic homogenized ones, and the finite element approximations related to the microscopic problem are defined to evaluate the homogenized coefficients subjected to periodic boundary conditions. Details of numerical implementation of homogenization magnetoelectric composite are briefed in Supplementary Information. Once the homogenized solutions for **u**^0^, *φ*^0^ and *ψ*^0^ are known then one can compute, the local displacement, potentials, fields, strains, stresses, and equivalent von-Mises stresses for each element and at each integration point. (The equivalent von-Mises stress at the nodes is an average of the values at the Gauss points of the surrounding elements with same material properties).

### Single-crystal FE–polycrystal FM composite

#### Application of electric field ${\mathbb{E}}$

**Figure 2:**
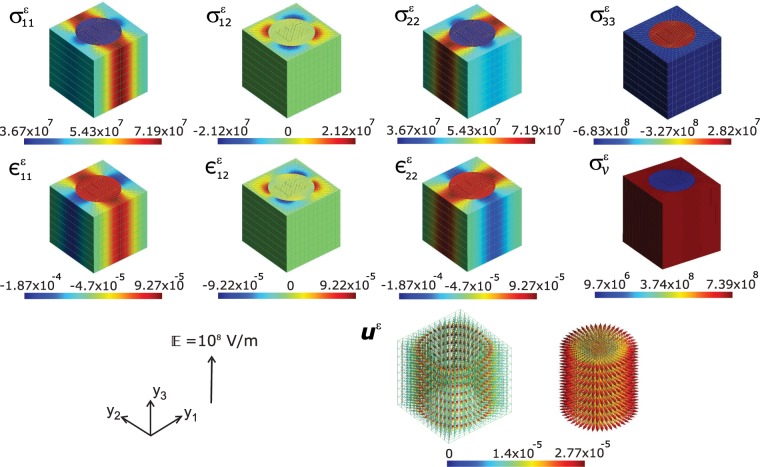
Map of local fields (computed at the nodal points of the FEM) of magnetoelectric composite BaTiO_3_–CoFe_2_O_4_, *viz*., the stress ${\sigma }_{ij}^{\varepsilon }(N/{m}^{2})$, strain ${\epsilon }_{ij}^{\varepsilon }$, the equivalent von Mises stress ${\sigma }_{v}^{\varepsilon }(N/{m}^{2})$, and displacement **u**^*ε*^(*m*) upon applying a global electric field ${\mathbb{E}}$ of 10^8^ *V*/*m* on the unit cell. Here the magnetic CoFe_2_O_4_ cylindrical pillars are surrounded by ferroelectric BaTiO_3_ matrix and both are aligned towards the y_3_ axis of the local coordinate system. Images drawn using Inkscape 0.92.4 (https://www.inkscape.org) under GNU General Public License and Gmsh version 2.15.0 (http://gmsh.info/) under GNU General Public License.

**Figure 3:**
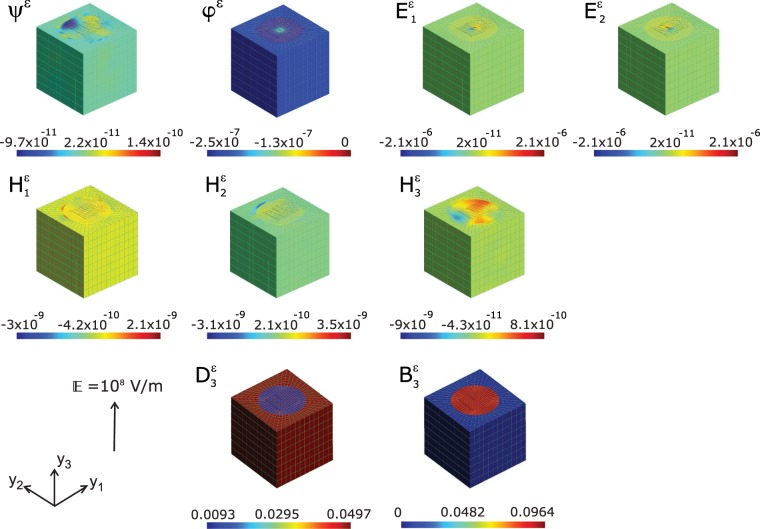
Map of local magnetic scalar potential *ψ*^*ε*^ (*A*), electric potential *φ*^*ε*^ (*V*), electric field ${{\rm{E}}}_{j}^{\varepsilon }(V/m)$, magnetic field ${{\rm{H}}}_{j}^{\varepsilon }(A/m)$, electric displacement ${{\rm{D}}}_{3}^{\varepsilon }(C/{m}^{2})$, and magnetic flux ${{\rm{B}}}_{3}^{\varepsilon }(Wb/{m}^{2})$ computed at the nodal points of the FEM, upon applying a global electric field ${\mathbb{E}}$ of 10^8^ *V*/*m* on a magnetoelectric composite of BaTiO_3_–CoFe_2_O_4_. Images drawn using Inkscape 0.92.4 (https://www.inkscape.org) under GNU General Public License and Gmsh version 2.15.0 (http://gmsh.info/) under GNU General Public License.

Here we model a system of 1–3 magnetoelectric composite consisting of a matrix of single-crystalline FE material holding an array of FM fibres inside it. As a classical system, we have chosen, CoFe_2_O_4_–BaTiO_3_, where BaTiO_3_ as FE and CoFe_2_O_4_ as the FM fibrous material. Either of these materials’ crystallites are chosen to experience no Euler rotation. i.e., the initial polarization of the BaTiO_3_ would be along the local *y*_3_–axis. Or the [001]–direction of the BaTiO_3_ coincides with the *y*_3_–axis of the local coordinate system. Consequently the unit cell of homogenization would be single-crystalline if at all we introduce physical properties corresponding to single crystalline FE and FM materials. Since we use, single crystalline data for BaTiO_3_ (from ref. ^42^) and polycrystalline data for CoFe_2_O_4_ (from ref. ^43^), the resulting system would be a single-crystal FE–polycrystal(or ceramic) FM composite. It is interesting to notice the distribution of microscopic fields computed at every nodal points of the FEM mesh constructing the unit cell. The distribution of local physical quantities across the microscopic material upon applying a global external electric field ${\mathbb{E}}=1{0}^{8}V/m$, which is large enough to saturate the electric response, is as shown in Figs. 2 and 3. Here the volume fraction of the FE matrix (BaTiO_3_) component in the ME composite is kept at *v*_*f*_ = 65% (or equivalently *v*_*f*_ of CoFe_2_O_4_ is 35%) conforming the ME coupling studies by Zheng *et al*.^19^ on vertically aligned nanostructures where CoFe_2_O_4_ nanopillars embedded epitaxially in BaTiO_3_.

When an electric field is applied to a ME composite, strain created by the piezoelectric BaTiO_3_ matrix couples to the embedded magnetostrictive CoFe_2_O_4_ pillars. Deformation of the material’s structure under ${\mathbb{E}}$ can result in modification of magnetic properties and vice versa. This phenomenon consequently alters the magnetization state of the magnetostrictive CoFe_2_O_4_ phase of the composite^44^. The state of stress within a body is determined when we know the values at each point of the six components of stress, *σ*_*ij*_ (computed using Supplementary Eq. (S15)). Thus we simulate the local stress field distribution through the CoFe_2_O_4_–BaTiO_3_ composite microstructure first. The results are depicted in Fig. 2. The off-diagonal shear stress components ${\sigma }_{13}^{\varepsilon }$ and ${\sigma }_{23}^{\varepsilon }$ are marginal, in this analysis. We observe longitudinal stress concentration in FE BaTiO_3_ matrix along *y*_2_ and *y*_1_ directions of the local coordinate system. In fact, the stress concentration is along a patch (brick red in ${\sigma }_{11}^{\varepsilon }$ and ${\sigma }_{22}^{\varepsilon }$) which spreads in three dimensions as is seen in Fig. 2. This leave a stress lowering along perpendicular directions in the microstructure (blue patches along FE matrix). Yet, another noticeable feature here is the reduced in-plane stress components ${\sigma }_{11}^{\varepsilon }$ and ${\sigma }_{22}^{\varepsilon }$ across the FM fibre of the ME composite. The shear component ${\sigma }_{12}^{\varepsilon }$ show a symmetric distribution with a tensile component (positive stress as is displayed in brick red patch) and compressive component (negative stress, the blue patch) acting diagonally across the composite plane. The distribution of the normal component ${\sigma }_{33}^{\varepsilon }$ shows tensile stress along the FM fibre (brick red) and a consequent compressive stress through FE matrix. This essentially results in relative contraction magnetic fibres along filed direction (*y*_3_–axis) confirming the magnetoelectric results on CoFe_2_O_4_ reported previously^45,46^. This contrasting distribution of the normal stress component precipitates in magnetization of the composite along the *y*_3_-axis. The equivalent von Mises stress computed at the nodal points express contrasting (Fig. 2) tensile behaviour of the composite unit cell. The ferroelectric BaTiO_3_ matrix shows relatively more tensile stress compared to the ferromagnetic CoFe_2_O_4_ fibre. This contrasting stress distribution in constituent phases of ME composite could precipitate in magnetization due to piezomagnetism. Electric-to-magnetic energy conversion can thus occur through the transmission of an electric field induced elastic stress from the ferroelectric phase to the magnetic phase. The occurrence of stress due to electric field, can in turn manifest as magnetization in FM phase of the composite (and this property is termed as *linear magnetostriction*)^5^. We have computed the average magnetization from Supplementary Eq. (S2) as $\langle {M}_{k}\rangle =\widetilde{{\alpha }_{ik}}/{\mu }_{0}\langle {E}_{i}\rangle$ where *μ*_0_ is the permeability of free space. The average value is computed to be 〈*M*_3_〉 ≈ 2.63 × 10^4^ (*A*/*m*) at the external electric field ${\mathbb{E}}=1{0}^{8}V/m$. 〈*M*_*k*_〉 components along *y*_1_ − *y*_2_–plane (or *xy*–plane of the ME composite) are zero as the magnetization is acting fully along the electric field direction.

This electric field induced magnetization indicates strong coupling between the two phases of the composite, since this value is less only by one order of magnitude than the magnetization **M** measured at external magnetic field (not at electric field as in the present study) in CoFe_2_O_4_–BaTiO_3_ composite multilayer thinfilms^19^. Electric field can bring about strain due to the piezoelectric effect intrinsic to BaTiO_3_. Hence the present result (along the out-of plane direction of the composite, along which the external field ${\mathbb{E}}$ is applied) indicates significant coupling between the magnetic and electric degrees of freedom in the composite. The strain distributions due to the impact of external electric field is shown in Fig. 2. No shear component including the ${\mathbb{E}}$ directions (i.e., *y*_3_–axis) appear in strains and therefore, the material planes including *y*_3_ do not suffer any shear deformation. The tensile strains *ϵ*_11_ and *ϵ*_22_ exhibit patterns complementing the corresponding stress distributions and so is the case with the in-plane shear strain component *ϵ*_12_. The displacement vector **u**^*ε*^ shown in figure explains how the crystallites are displaced in the microstructure. In order to discern the displacement vector directions clearly we show the displacements of the FM fibre and the FE matrix separately. In both phases of the composite the displacement vectors are pointing outwards in-plane. This explains why the normal strain component *ϵ*_33_ is absent in the unit cell. The applied voltage ${\mathbb{E}}$ on the composite generate strain in the piezoelectric BaTiO_3_ which alters the magnetic state of the symmetrically attached magnetic material CoFe_2_O_4_ via elastic coupling and resulting in the precipitation of magnetization.

Any problem with specified charges can be solved by computing the electric potential and thereby obtaining the electric field by taking the derivative of the potential using the Supplementary Eq. (S6). Similar is the case with magnetic field computed from the derivative of the magnetic potential, though one cannot have equivalent “magnetic charges” that are responsible for the field since moving electrical charges creates magnetism in a material. The distribution of magnetic and electric potential are shown in Fig. 3. This essentially provides a measure of the spatial distribution of the corresponding fields which are the derivatives of these scalar fields. The generation of magnetic potential due to the external electric field ${\mathbb{E}}$ would provide the key to the underlying movement of electric charges responsible for the precipitation of magnetic dipoles and thereby the magnetization. Here we see the distribution of magnetic and electric potential are uniform throughout the ferroelectric matrix which essentially carves out equipotential surfaces in this phase. Since electric and magnetic fields are vector quantities, each of their components ${{\rm{E}}}_{{\rm{j}}}^{\varepsilon }$ and ${{\rm{H}}}_{{\rm{j}}}^{\varepsilon }$ are shown in Fig. 3. Here we have not shown the component ${{\rm{E}}}_{3}^{\varepsilon }$ as it is the same as the applied field ${\mathbb{E}}$ everywhere in the unit cell. The electric displacement vector **D** is expressed as a linear combination of polarization **P** and electric field **E** by **D** = *κ*_0_**E** + **P**, where *κ*_0_ is the permittivity of free space. Hence the local electrical displacement field ${{\rm{D}}}_{3}^{\varepsilon }$ shown in Fig. 3 explicitly reveals the polarization **P** direction to be along the *y*_3_–axis direction. The polarization orientation is underscored by the marginal values of the components ${{\rm{D}}}_{1}^{\varepsilon }$ and ${{\rm{D}}}_{2}^{\varepsilon }$. The magnetic flux density ${{\rm{B}}}_{3}^{\varepsilon }$ shown in Fig. 3 indicates the distribution density of induced magnetic field strength on the microstructure. The ferroelectric BaTiO_3_ matrix is practically devoid of the presence of magnetic induction while the magnetic CoFe_2_O_4_ contains flux of magnetic field. The presence of magnetic flux density in the FM fibre indicates that the magnetic domains in the material (CoFe_2_O_4_) get aligned along the *y*_3_–axis which due to an induced magnetic field and thus be concluded that the magnetic easy axis is along *y*_3_–axis.

**Figure 4:**
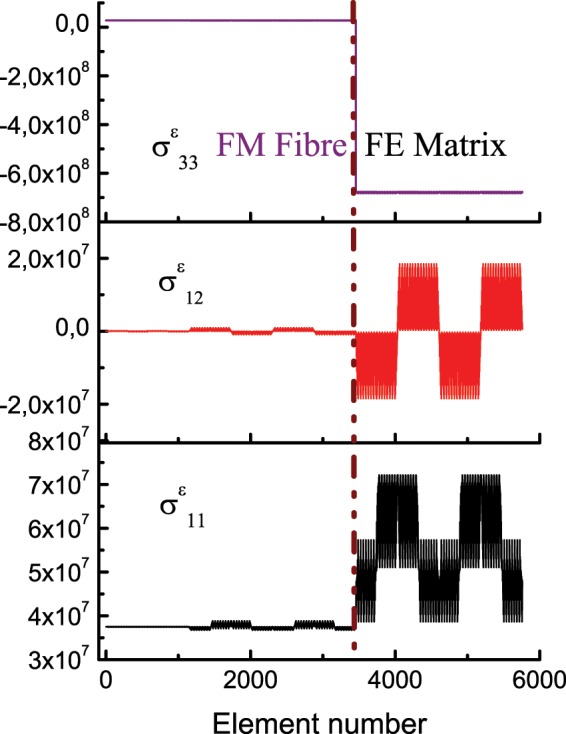
Plot of local stresses ${\sigma }_{ij}^{\varepsilon }(N/{m}^{2})$ acting at each grain (finite element) of the composite microstructure of BaTiO_3_–CoFe_2_O_4_ upon applying a global electric field ${\mathbb{E}}$ of 10^8^*V*∕*m* on the unit cell.

The elemental averages of local fields computed from the nodal values for each element of the FEM of the unit cell are shown in Supplementary Fig. S2. This is to show the distribution patterns pertaining to each grains/crystallites in the microstructure of the composite as we are assuming that each finite element surrogates the grains constituting the polycrystal. We have plotted the elemental averages of stress in Fig. 4 in order to see how it changes inside the microstructure. 3456 grains (finite elements in the model) constitute the magnetic fibre out of the total 5760 finite elements in the unit cell for this analysis. It can be discerned that both the normal components ${\sigma }_{ii}^{\varepsilon }$ and the shear component ${\sigma }_{12}^{\varepsilon }$ shows abrupt changes at the interface of the composite (i.e., at the element number 3456 corresponding to the boundary of the fibre-matrix phase). And inside the matrix both ${\sigma }_{11}^{\varepsilon }$ and ${\sigma }_{12}^{\varepsilon }$ varies. The shear term ${\sigma }_{12}^{\varepsilon }$ oscillates between negative (contractive) and positive (tensile) values at regular intervals while ${\sigma }_{11}^{\varepsilon }$ varies between a lower (lesser tensile stress) and upper bound (higher tension). It would be interesting to see the corresponding strain curves of this analysis as it is shown in Supplementary Fig. S3.

#### Application of magnetic field ${\mathbb{H}}$

**Figure 5:**
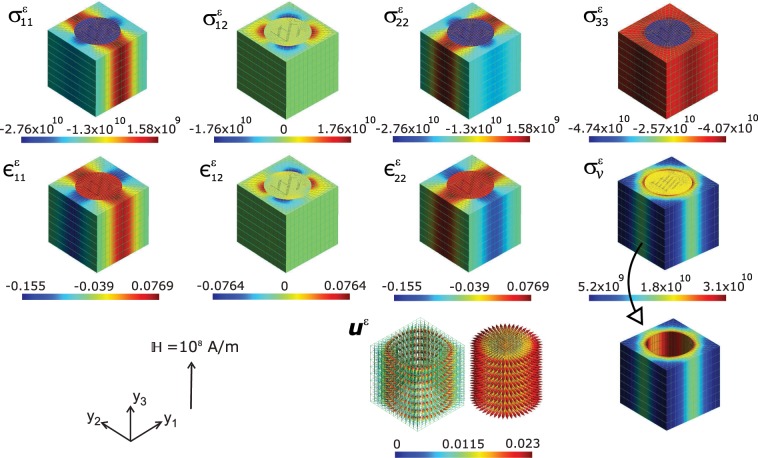
Map of local fields (computed at the nodal points of the FEM) of magnetoelectric composite BaTiO_3_–CoFe_2_O_4_, *viz*., the stress ${\sigma }_{ij}^{\varepsilon }(N/{m}^{2})$, strain ${\epsilon }_{ij}^{\varepsilon }$, the equivalent von Mises stress ${\sigma }_{v}^{\varepsilon }(N/{m}^{2})$, and displacement **u**^*ε*^(*m*) upon applying a biasing magnetic field ${\mathbb{H}}$ of 10^8^*A*/*m* on the unit cell. Here the magnetic CoFe_2_O_4_ cylindrical pillars are surrounded by ferroelectric BaTiO_3_ matrix and both are aligned towards the y_3_ axis of the local coordinate system. Images drawn using Inkscape 0.92.4 (https://www.inkscape.org) under GNU General Public License and Gmsh version 2.15.0 (http://gmsh.info/) under GNU General Public License.

**Figure 6:**
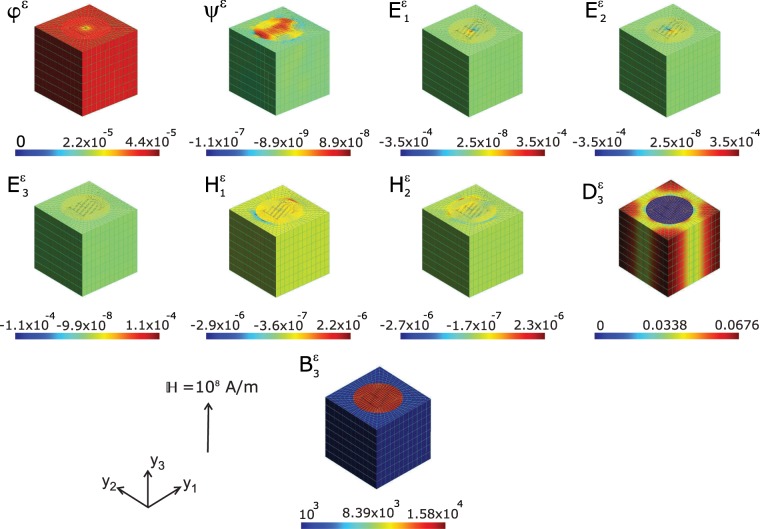
Map of local electric potential *φ*^*ε*^(*V*), magnetic scalar potential *ψ*^*ε*^(*A*), electric field ${{\rm{E}}}_{j}^{\varepsilon }(V/m)$, magnetic field ${{\rm{H}}}_{j}^{\varepsilon }(A/m)$, electric displacement ${{\rm{D}}}_{3}^{\varepsilon }(C/{m}^{2})$, and magnetic flux ${{\rm{B}}}_{3}^{\varepsilon }(Wb/{m}^{2})$ computed at the nodal points of the FEM, upon applying a biasing magnetic field ${\mathbb{H}}$ of 10^8^*A*/*m* on a magnetoelectric composite of BaTiO_3_–CoFe_2_O_4_. Images drawn using Inkscape 0.92.4 (https://www.inkscape.org) under GNU General Public License and Gmsh version 2.15.0 (http://gmsh.info/) under GNU General Public License.

Since the application of a magnetic field can modify the electric polarization and create electrical response in magnetoelectrics, instead of electric field we now apply an external magnetic field ${\mathbb{H}}$ to the composite and simulate the impact of it on the microstructure. The biasing magnetic field applied here would be sufficient to saturate the magnetic phase in the ME composite. The results are plotted in Figs. 5 and 6. The distributions shown in Fig. 5, offers an outline of the mechanical response of the ME composite CoFe_2_O_4_– BaTiO_3_ consequent to the external magnetic field ${\mathbb{H}}$. There is an interplay of piezomagnetism coupled with piezoelectricity for this kind of a phenomenon to occur in this material. The elastic deformation is generated by magnetostriction and the resulting strain is transmitted to the piezoelectric material, resulting in an induced electric polarization due to piezoelectric effect. The local stress distributions corresponding to the normal components ${\sigma }_{11}^{\varepsilon }$ and ${\sigma }_{22}^{\varepsilon }$ show a clear contraction across FM fibre comparing to the FE matrix. Yet, the normal component ${\sigma }_{33}^{\varepsilon }$ acting along *y*_3_–axis shows that the compressive stress along the fibre axis (*y*_3_–axis) is pronounced than that along the matrix. This is in conformity with the magnetostriction (*λ*) analysis in the magnetic CoFe_2_O_4_, where the magnetic field give rise to contraction of the CFO nanopillars (*λ*_∥_ < 0) along the applied magnetic field (${\mathbb{H}}$) direction^45,46^. At the same time these analyses observe that the magnetostriction- which essentially is a measure of the magnetic field induced strain- in the *xy*-plane of CFO (*λ*_⊥_ > 0). Consequently, the inplane strains ${\epsilon }_{11}^{\varepsilon }$ and ${\epsilon }_{22}^{\varepsilon }$ should be tensile as it is correctly observed in Fig. 5. The inplane shear components of stress and strain (${\sigma }_{12}^{\varepsilon }$ and ${\epsilon }_{12}^{\varepsilon }$) show a zero-sum distribution meaning the shear across one plane is offset by the same across the opposite plane. The von Mises distribution corresponding to the FE matrix is sliced apart on Fig. 5 bottom panel to highlight the stress concentration at the boundary between the FE and FM phases of the ME composite.

Fig. 6 shows the distributions corresponding to electrical and magnetic field variables consequent to an applied magnetic field ${\mathbb{H}}=1{0}^{8}A/m$. There is significant presence of electric potential *φ*^*ϵ*^ throughout the composite microstructure indicating magnetic-field induced electrical charge distribution in the ME composite. The electric potential generated by the external magnetic field is distributed nearly uniformly across the magnetic and ferroelectric phases of the composite. Moreover, the value is positive in contrast to the case when external electric field loading (see Fig. 3) where the values are negative and two orders of magnitude less in absolute values. One can infer from this observation that the polarity of the charges generated by the application of ${\mathbb{E}}$ and ${\mathbb{H}}$ fields are opposite in kind and differs drastically in its density. The magnetic potential *ψ*^*ε*^ distribution in response to ${\mathbb{H}}$ also exhibits contrasting profile with respect to the distribution at external electric field ${\mathbb{E}}$. While at ${\mathbb{H}}$ field loading the FE matrix display a negative magnetic potential accumulation, the corresponding distribution is positive in the case of ${\mathbb{E}}$ field loading (see Fig. 3). Nevertheless, the strength of the magnetic potential distribution across the the FE matrix is greater by one order of magnitude in the case of ${\mathbb{H}}$ field loading. In the FM fibre however, in both cases we see an overlap of negative and positive magnetic potential distribution though the magnitudes are greater in ${\mathbb{H}}$ loading. Another numerical experiment with an in-plane magnetic field ${{\mathbb{H}}}_{x}=1A/m$ and volume fraction *v*_*f*_ = 0.2 of CFO fibre is conducted to compare the local displacement, electric and magnetic potential of the present model with that of Kuo and Bhattacharya^47^. The results which are given in Supplementary material (Supplementary Fig. S7) are in good accord with that of Kuo and Bhattacharya.

The local magnetic fields computed reflected these contrasts very well, as is observed in Fig. 3, where the direction of the out of plane local field ${{\rm{H}}}_{3}^{\varepsilon }$ is negative in the FE matrix while it is equal to the external field in the ${\mathbb{H}}$ loading (This is not shown here as it is uniform value of 10^8^*A*∕*m* throughout the microstructure). The electric displacement component along the *y*_3_–axis ${{\rm{D}}}_{3}^{\varepsilon }$ shown in Fig. 6 is a measure of the electrical polarization due to the impact of electric field in a material. (The other two vector components of ${{\rm{D}}}_{{\rm{j}}}^{\varepsilon }$ are negligible compared to ${{\rm{D}}}_{3}^{\varepsilon }$ and hence are not shown). Yet, here the generation of electric displacement is a consequence of magnetic field ${\mathbb{H}}$ applied externally. The magnetic-field-induced stress/strain in the magnetostrictive CoFe_2_O_4_ phase will be partly transferred to the piezoelectric BaTiO_3_ via the bounding interfaces, leading to an induced electric polarization **P** due to the piezoelectric effect. The average of polarization at constant magnetic field can be computed using Eq. (S3), as $\langle {P}_{k}\rangle =\widetilde{{\alpha }_{ik}}\langle {H}_{i}\rangle$ using homogenized values of the ME coupling $\widetilde{{\alpha }_{ik}}$ obtained from homogenization program. The average polarization along the *y*_3_–axis (out-of plane direction of the composite) thus obtained here is P_3_ ≈ 0.033*C*∕*m*^2^ (see Table 1). This is one order of magnitude less than the saturation polarization (P_s_ = 0.23*C*∕*m*^2^) measured at electric field and normalized to the volume fraction of BaTiO_3_ in the planar multilayer thinfim assembly of CoFe_2_O_4_ nanopillars embedded in BaTiO_3_ matrix^19^. Thus the magneto-electrical coupling in the present architecture is so prominent as the present value of electrical polarization is a direct consequence of magnetic field. It may be noticed that the P_3_ obtained here is induced by the magnetic field unlike in ref. ^19^ where the polarization curve is measured at external electric field. Nonetheless, the absence of electric displacement in the magnetic phase (blue) indicates that electric polarization of the composite is that of the underlying FE constituent material in the ME composite. The magnetic flux ${{\rm{B}}}_{3}^{\varepsilon }$ along *y*_3_–axis (here also the other vector components are marginal) shows a uniform distribution both along fibre and the matrix though with different values. Here we can see that magnetic field permeates through the entire material as a consequence of the external ${\mathbb{H}}$.

**Table 1:** Average values of electric displacement 〈D_3_〉 (*C*/*m*^2^), magnetic flux 〈B_3_〉(*Wb*/*m*^2^), magnetization 〈M_3_〉(*A*/*m*), and electric polarization 〈P_3_〉(*C*/*m*^2^) of 1–3 magnetoelectric composite of single crystal BaTiO_3_–ceramic CoFe_2_O_4_ at external electric (${\mathbb{E}}=1{0}^{8}V/m$) and magnetic fields (${\mathbb{H}}=1{0}^{8}A/m$).

Microstructure	*v*_*f*_ (%)	at electric field ${\mathbb{E}}$			at magnetic field ${\mathbb{B}}$		
		〈D_3_〉	〈B_3_〉	〈M_3_〉(×10^4^)	〈D_3_〉	〈B_3_〉(×10^3^)	〈P_3_〉
Customary	20	0.042	0.022	1.75	0.022	3.95	0.022
	35	0.036	0.033	2.63	0.033	6.15	0.033
	50	0.030	0.039	3.07	0.039	8.37	0.039
	60	0.026	0.030	2.35	0.030	9.85	0.030
Honeycomb	20	0.042	0.020	1.62	0.020	3.93	0.022
	35	0.036	0.030	2.32	0.030	6.12	0.033
	50	0.030	0.033	2.61	0.033	8.32	0.039
	60	0.026	0.032	2.58	0.030	9.78	0.030

#### Varying concentration of the FE component

**Figure 7:**
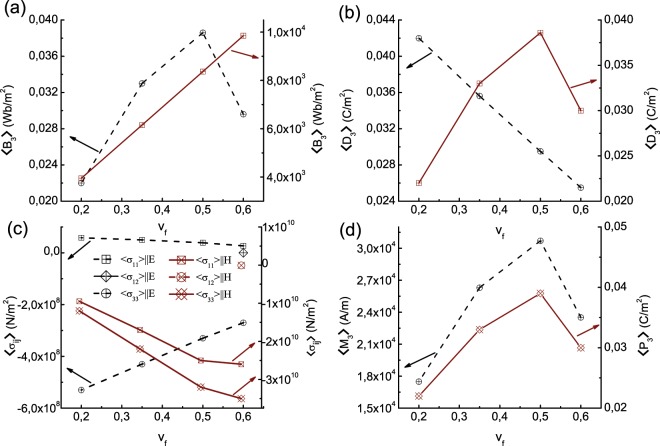
Plots of the average (**a**) magnetic flux 〈B_3_〉(*Wb*/*m*^2^), (**b**) electric displacement 〈D_3_〉(*C*/*m*^2^), (**c**) stress 〈σ_*ij*_〉(*N*/*m*^2^), (**d**) the magnetization 〈M_3_〉(*A*/*m*) and polarization 〈P_3_〉(*C*/*m*^2^) upon applying a biasing electric field (${\mathbb{E}}$) (dashed line plots) and magnetic field (${\mathbb{H}}$) (line plots) separately on magnetoelectric composite of BaTiO_3_–CoFe_2_O_4_ at various volume fractions (*v*_*f*_) of the FM fibrous material CoFe_2_O_4_ of the composite.

The content of either the FM or FE component can impact the distribution and generation of local and for that matter the average fields of the ME composite. ME property dependence on constituent volume fraction was demonstrated previously in many studies^29^. To explore this particular aspect, the volume fraction (*v*_*f*_) of the FM fibre, CoFe_2_O_4_ is varied and the local (microscopic) fields and the property averages are computed and are given in Fig. 7. (The local field distributions at every concentration of CoFe_2_O_4_ is not presented for brevity). Average values of stress 〈*σ*_*ij*_〉, magnetic flux density 〈B_*j*_〉, and electric displacement 〈D_*j*_〉 are computed using Supplementary Eqs. (S18–S20). Average magnetization and polarization are computed using $\langle {{\rm{M}}}_{{\rm{k}}}\rangle =\widetilde{{\alpha }_{ik}}/{\mu }_{0}\langle {{\rm{E}}}_{{\rm{i}}}\rangle ,\ {\rm{and}}\ \langle {{\rm{P}}}_{{\rm{k}}}\rangle =\widetilde{{\alpha }_{ik}}\langle {{\rm{H}}}_{{\rm{i}}}\rangle$ respectively. Computation of 〈*σ*_*ij*_〉, 〈B_*j*_〉, 〈D_*j*_〉, 〈M_k_〉 and 〈P_k_〉 are made from the knowledge of homogenized magnetic–electro–elastic coefficients *viz*., ${\widetilde{C}}_{ijkl}^{EH}$, ${\widetilde{e}}_{ijk}$, ${\widetilde{e}}_{kij}^{M}$, ${\widetilde{\kappa }}_{ij}^{\epsilon H}$, and ${\widetilde{\alpha }}_{ij}$, and the averages of strain, electric and magnetic fields *viz*., $\frac{\partial {u}_{j}^{0}({\bf{x}})}{\partial {x}_{k}}$, $\frac{\partial {\phi }^{0}({\bf{x}})}{\partial {x}_{j}}$ and $\frac{\partial {\psi }^{0}({\bf{x}})}{\partial {x}_{j}}$ respectively derived from homogenization. The averages vary significantly with volume fraction as in the picture. The components of averages along the *xy*–plane of the ME composite are marginal except the emergence of the in-plane shear component of stress 〈*σ*_12_〉 at the 50% volume fraction of CoFe_2_O_4_ (eg. Fig. 7(c)). At the same volume fraction *v*_*f*_ = 50% of the FM component (which means 100 × (1 − *v*_*f*_) = 50% of the FE constituent as well) of the ME composite, 〈D_3_〉, 〈M_3_〉 and 〈P_3_〉 peak. The normal stress component 〈*σ*_33_〉 along the *y*_3_–axis of the composite (i.e., along the out-of-plane direction) in both cases of electric and magnetic biasing in Fig. 7(c) is compressive (negative) in accordance with the conclusions from the magnetostriction being negative along the field direction in CoFe_2_O_4_^45,46^. The electric displacement 〈D_3_〉 shows contrasting progression with the content of CoFe_2_O_4_ (Fig. 7). While the $\langle {{\rm{D}}}_{3}\rangle \parallel {\mathbb{E}}$ is decreasing sharply with the *v*_*f*_ of CoFe_2_O_4_, the $\langle {{\rm{D}}}_{3}\rangle \parallel {\mathbb{H}}$ increases first and peaks at *v*_*f*_ = 50% and then declines. This implies the development of polarization charges with the addition of magnetic content consequent to a magnetic field in the composite. Similar is the case with magnetic flux density $\langle {{\rm{B}}}_{3}\rangle \parallel {\mathbb{H}}$ given in Fig. 7(a) where it strengthens with *v*_*f*_. Nonetheless, $\langle {{\rm{B}}}_{3}\rangle \parallel {\mathbb{E}}$ is very feeble in magnitude compared to the $\langle {{\rm{B}}}_{3}\rangle \parallel {\mathbb{H}}$ at magnetic field ${\mathbb{H}}$.

### Honeycomb microstructure

Here we study the local and global fields using a honeycomb lattice unit cell of the ME composite in order to explore the robustness of the POSTMAT developed for this study. The results of the local fields are summarised in Supplementary Figs. S4 and S5 consequent to the application of external electric (${\mathbb{E}}$) and magnetic (${\mathbb{H}}$) fields respectively. The stress/strain scales are similar in both cases of honeycomb and customary fibre reinforced lattices. Here too it can be seen that the normal stress ${\sigma }_{33}^{\varepsilon }$ passing through the FM fibres are more compressive than that through the FE matrix in accordance with the negative magnetostriction observed in CoFe_2_O_4_ along the axis of magnetic field (Supplementary Fig. S5). The inplane components of electromagnetic averages 〈D_k_〉, 〈B_k_〉, 〈M_k_〉 and 〈P_k_〉 are absent indicating the electrical and magnetic domain alignment along the applied field direction i.e., the *y*_3_–axis. Essentially, it points out that the fields applied are able to saturate the composite.

The average values computed using honeycomb microstructure for BaTiO_3_–CoFe_2_O_4_ composite is plotted in Supplementary Fig. S6, where the content of magnetic fibre CoFe_2_O_4_ is 35% of the total volume as in the case of averages plotted in Fig. 7. The average stress 〈*σ*_ij_〉, electric displacement 〈D_3_〉, magnetic flux density 〈B_3_〉, magnetization 〈M_3_〉 at external electric field (${\mathbb{E}}$), and electric polarization 〈P_3_〉 at external magnetic field (${\mathbb{H}}$) shown in Supplementary Fig. S6 exhibit the same trends as in the case of customary fibre-reinforced 1–3 ME composite (Fig. 7) and same values except for magnetization. The average values computed using the customary fibre reinforced and honeycomb 1–3 microstructure are summarised in Table 1 for numerical comparison. The values in general are similar except for the cases of magnetic parameters, magnetization 〈M_3_〉 and magnetic flux 〈B_3_〉, wherein the largest deviation of  ≈16–17% occurs at a volume fraction 50% of the magnetic CoFe_2_O_4_.

## Conclusions

In this paper we analyse the distribution of microscopic fields in a fibre-reinforced magnetoelectric composite of 1–3 connectivity due to the application of external electric and magnetic fields. Macroscopic averages of the resulting, elastic, electric and magnetic responses (i.e., mechanical stress, electric displacement and magnetic flux density) are computed from the knowledge of homogenized macroscopic degrees of freedom. A homogenization method combined with a variational formulation is developed for a periodic multiferroic magnetoelectric composite of lowest crystallographic symmetry of the constituent phases through a two-scale asymptotic analysis of the microscopic electric, magnetic and mechanical fields to characterise the impact on each of them of an external field. Here the interface between the constituent FE and FM phases are assumed to be ideal and semi-coherent which facilitates transfer of strain/stress across the boundary. The methodology is implemented numerically by modifying the software POSTMAT (*material postprocessing*). As an example system, the ferri/ferromagnetic CoFe_2_O_4_ fibres embedded in ferroelectric BaTiO_3_ matrix is chosen as a typical magnetoelectric composite for simulation. The BaTiO_3_ phase is treated as single crystalline and the CoFe_2_O_4_ phase as polycrystalline in this simulation. In order to ascertain the robustness of the model algorithm, we used two representative unit cells to surrogate the 1–3 configuration *viz*., the customary fibre-reinforced composite architecture and the honeycomb lattice. The microscopic fields (or *local fields*) pertaining to the electrical, magnetic, and spatial degrees of freedom and their coupling interactions at any arbitrary point in the unit cell is obtained. A convergence analysis of magnetoelectric properties with unit cell size allows us to determine the simulation-space independent, equivalent magnetoelectric properties of the composite.

An electric field (${\mathbb{E}}$ parallel to *y*_3_–axis) sufficient to saturate the material electrically is first applied to the material and the resulting local fields are mapped alongwith the averages. Electric-to-magnetic energy conversion can plausibly be occur through the transmission of an electric field induced elastic stress from the ferroelectric phase to the magnetic phase. Significant impact of coupling between the magnetic and electric degrees of freedom in the composite is obtained from this analysis. Distribution of magnetic and electric potential (they are important as it provides a measure of the spatial distribution of the corresponding fields that are derivatives of these scalar fields) are found to be uniform throughout the ferroelectric matrix which essentially carves out equipotential surfaces in this phase. Average magnetization 〈M_k_〉, is computed and found to be within reasonable range measured in similar systems. The magnetization 〈M_3_〉 is found to be along the direction of the applied field which is the out-of-plane direction of the ME composite.

Strong magnetoelectric coupling can result in an applied magnetic field (${\mathbb{H}}$ parallel to *y*_3_–axis) as well by the confluence of magnetostrictive effects and piezoelectricity to achieve an electric response. Generation of electrical polarization along the field direction is underscored by the appearance of local electrical displacement ${{\rm{D}}}_{3}^{\varepsilon }$ with marginal values of ${{\rm{D}}}_{1}^{\varepsilon }$ and ${{\rm{D}}}_{2}^{\varepsilon }$. There is an interplay of piezomagnetism coupled with piezoelectricity for this kind of phenomenon to occur in CoFe_2_O_4_–BaTiO_3_ composite. The elastic deformation is generated by magnetostriction of the FM phase and the consequent strain is permeated to the FE material, resulting in an induced electric polarization due to piezoelectric effect. The presence of magnetic flux density ${{\rm{B}}}_{3}^{\varepsilon }$ in the CoFe_2_O_4_ fibre indicates that the magnetic domains in the FM material get aligned along the *y*_3_–axis due to magnetic field and thus be concluded that the magnetic easy axis passes through *y*_3_–axis. The normal stress component ${\sigma }_{33}^{\varepsilon }$ acting along *y*_3_–axis reveals that the compressive stress (because of its negative value) along the fibre (*y*_3_–axis) is pronounced than that along the matrix. This is in conformity with the magnetostriction (*λ*) analyses in the magnetic CoFe_2_O_4_, where the magnetic field give rise to contraction of the CFO (*λ*_∥_ < 0) along the applied magnetic field (${\mathbb{H}}$) direction^45,46^. The electric potential generated by the external magnetic and electric fields (Figs. 3 and 6) are distributed nearly uniformly across the magnetic and ferroelectric phases of the composite showing that the polarity of electric charges generated by the application of ${\mathbb{E}}$ and ${\mathbb{H}}$ fields are opposite in kind and differs drastically in its density. Average polarization 〈P_3_〉 resulted from external magnetic field is computed and found to be one order of magnitude less than **P** measured in similar systems but measured at biasing electric field manifesting strong cross coupling of electricity and magnetism.

Simulation varying the content of magnetic phase exhibits magnetic and electrical parameters peak at equal volume fraction of both the phases of the ME composite. The POSTMAT (*material postprocessing*) and the preceding procedure presented in this study would enable a holistic study on the overarching impact of elastic, electric and magnetic fields on multiferroic magnetoelectric materials. The insights obtained from the present study will provide a basis for extending the application of systematic design procedures in finely resolved scales to new and challenging materials for real-world electronic applications.

## Supplementary information

Supplementary Information.
