# Supplementary material for: Homogenization method for microscopic characterization of the composite magnetoelectric multiferroics
Source: Sci Rep. 2020 Jan 28;10:1276. doi: 10.1038/s41598-020-57977-w (PMC6987106; doi:10.1038/s41598-020-57977-w)
Supplement: Supplementary file 1 — Supplementary Information. [file 41598_2020_57977_MOESM1_ESM.pdf]

Supplementary Information to Homogenization method for microscopic characterization of the composite magnetoelectric multiferroics

K.P. Jayachandran,* J.M. Guedes, and H.C. Rodrigues
IDMEC, Instituto Superior Técnico, Universidade de Lisboa, Lisboa, Av. Rovisco Pais, 1049-001 Lisbon , Portugal

This supporting material for the work on homogenization method for microscopic characterization of the composite magnetoelectric multiferroics include Literature review, theory of homogenization, its numerical implementation and few key results especially with respect to the honeycomb microstructure.

I. INTRODUCTION

The materials classified as magnetoelectric multiferroics possess both the magnetic and ferroelectric orders[1]. More than having them make it to exploit the functionalities of both the orders, a coupling between the ferromagnetic and FE states enable appearance of novel properties not present in either of the states[2]. Simultaneous occurrence of magnetism and ferroelectricity in multiferroic materials is constrained by the conflicting physical requirements of partially filled d orbitals for magnetism and empty d orbitals for ferroelectricity to manifest[3,4]. These restrictions results in rare occurrence of intrinsic multiferroics.

Nevertheless, substantial ME effect can be derived through fabricating composites of a ferroelectric (FE) and a ferromagnetic (FM) material in the form composites[5]. Crystallites of the two phases are assumed to be in good mechanical contact if the two phases are polycrystalline. The FE phase is poled near the ferroelectric Curie temperature (T_C) in a strong electric field to make the composite piezoelectric while the magnetic poling of the FM phase is accomplished in a similar way, by annealing the composite in a magnetic field near the Néel temperature (T_N). When an electric field is applied to this composite the FE grains elongate parallel to the electric field. The change in shape of the ferroelectric grains causes the ferromagnetic grains to deform, resulting in a change in magnetization[6]. The connectivity too play an important role in property development in multiphase solids where the connection patterns can change some physical properties[7].

Inclusion of FM nanoparticles into a FE matrix to form bulk particulate composites, where the interfacial strain is transmitted through the grain boundaries constitute one of this routes[8]. Another important way is stacking of FM and FE sheets into composite magnetoelectric laminate where the strain is transmitted between phases relying on the bonding between the layers[9]. Since the voltage control of magnetism in magnetic metals are hampered by the short screening lengths, researchers began to explore ultra thinfilms[10] and magnetic metals/ferroelectric oxide bilayers[11,12]. Electrostatic doping can alter the FE–FM interface and the magnetoelectric effect can penetrate deeper than the screening length into the metallic $La_{0.67}Sr_{0.33}MnO_3$ (LSMO) in a LSMO–BTO heterostructure[13]. Single crystal ferroelectric substrates are being used as FE–FM systems for utilizing the anisotropy and symmetry to its full potential in planar geometry in multilayer thinfilms. The magnetic field induced electric polarization (direct magnetoelectric coupling) is so vital in device applications that the magnetic field-produced strain in a magnetic phase transferring into a piezoelectric phase and inducing a charge output, is the main working mechanism of magnetic sensing[14], and also perceived as a criterion for magnetic detection. Burton and Tsymbal show that magnetic reconstruction induced by switching of electric polarization can be facilitated by the doping of divalent cation near the magnetic phase transition[15]. Uetsuji et al., have studied the coupling mechanical response consequent to the application of a magnetic field in magnetoelectric laminated composite[16].

II. THEORY

A. Constitutive equations and homogenization

The magnetoelectric effect is described by a term in the thermodynamic potential $\Phi_{\mathbf{me}}$ that is linear both in the magnetic ($\mathbf{H}$) and electric ($\mathbf{E}$) field, such that

$$\Phi_{\mathbf{me}} = -\alpha_{ik} E_i H_k \quad \text{(S1)}$$

where the magnetoelectric coupling coefficient α_{ik} is an unsymmetrical tensor[17]. When $\mathbf{H} = 0$, the

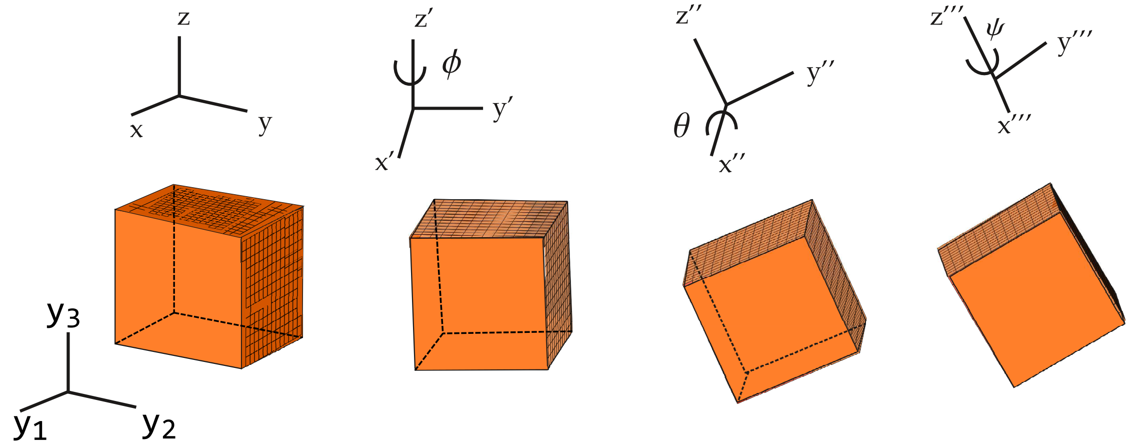

Figure S1. The successive angles of anti-clockwise rotations subjected to the crystallites (grains) on the ferroelectric phase of the ME composite as defined by the Euler angles ϕ°, θ° and ψ°. Here $(y_1, y_2, y_3) \equiv \mathbf{y}$ are the local and $(x, y, z) \equiv \mathbf{y}'$ are the crystallographic coordinate systems. Images drawn using Inkscape 0.92.4 (https://www.inkscape.org) under GNU General Public License and Gmsh version 2.15.0 (http://gmsh.info/) under GNU General Public License.

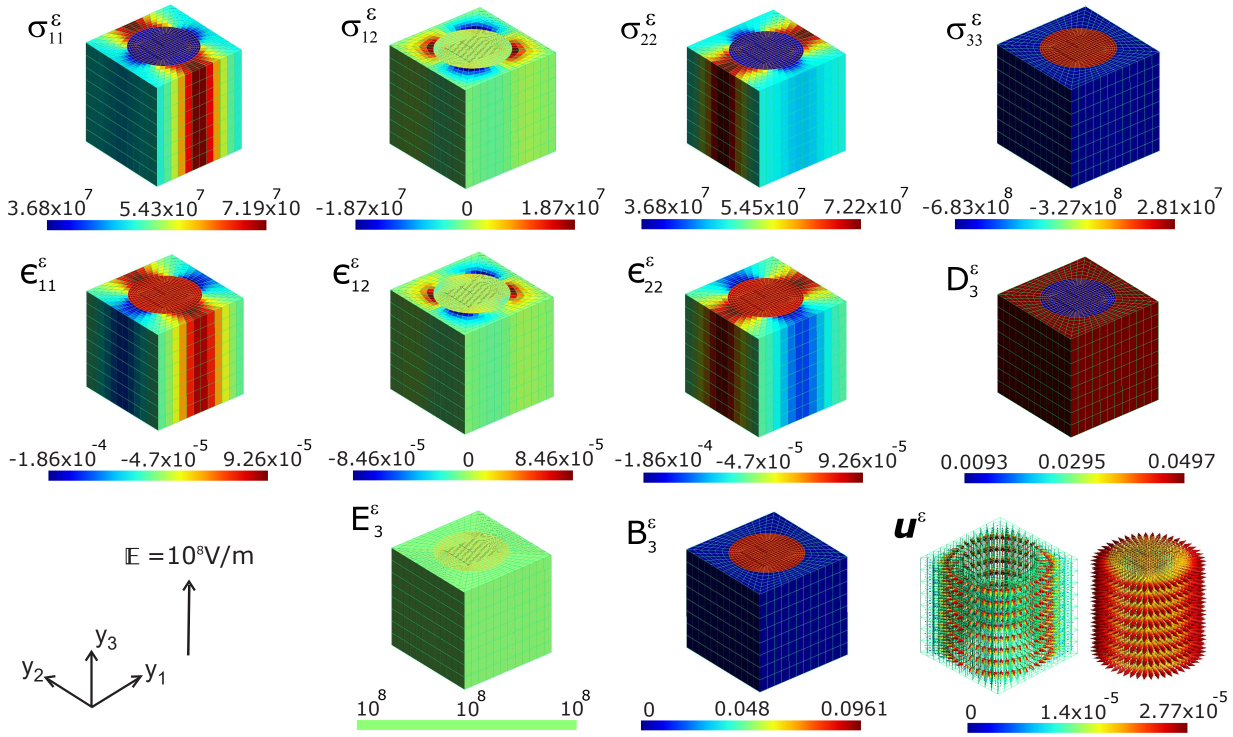

Figure S2. Map of local fields (averaged at the elements of the FEM) of magnetoelectric composite $BaTiO_3$–$CoFe_2O_4$, *viz.*, the stress $\sigma^{\varepsilon}_{ij}$ (N/m^2), strain $\epsilon^{\varepsilon}_{ij}$, the electrical displacement $D_3{}^{\varepsilon}$ (C/m^2), the electrical field $E_3{}^{\varepsilon}$ (V/m), the magnetic flux density $B_3{}^{\varepsilon}$ (Wb/m^2) and the material displacement $\mathbf{u}^{\varepsilon}$ (m) upon applying a global electric field $\mathbb{E}$ of $10^8 V/m$ on the unit cell. Here the polycrystalline magnetic $CoFe_2O_4$ cylindrical pillars are surrounded by single crystalline ferroelectric $BaTiO_3$ matrix and both are aligned towards the y_3 axis of the local coordinate system. Images drawn using Inkscape 0.92.4 (https://www.inkscape.org) under GNU General Public License and Gmsh version 2.15.0 (http://gmsh.info/) under GNU General Public License.

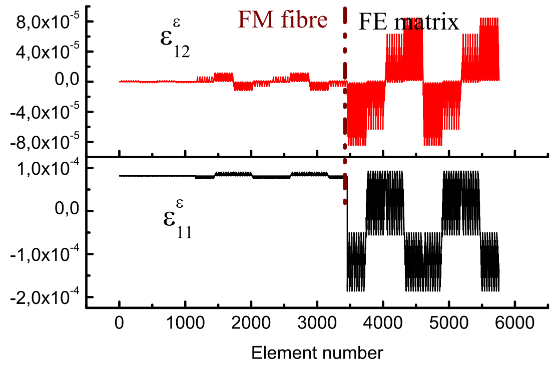

Figure S3. Plot of local strains $\epsilon^{\varepsilon}_{ij}$ acting at each grain (finite element) of the composite microstructure of $BaTiO_3$–$CoFe_2O_4$ upon applying a global electric field $\mathbb{E}$ of $10^8 V/m$ on the unit cell.

electric field generates a magnetization

$$M_k = \alpha_{ik} E_i \tag{S2}$$

and when $\mathbf{E} = 0$, the magnetic field generates an electrical polarization

$$P_i = \alpha_{ik} H_k \tag{S3}$$

A two-scale asymptotic homogenization analysis combined with a variational formulation is developed for determination of the equivalent material properties of a periodic multiferroic magnetoelectric composite. Local and average (global) electrical, magnetic, and mechanical constitutive behaviour are computed. The model takes into account the ferroelectric and ferromagnetic, and the composite magnetoelectric phases by treating the constitutive behaviour. For a linear magneto-electro-elastic solid, constitutive equations are governed by the electrical, mechanical and magnetic fields. The model quantifies the local electrical and magnetic potential, displacements, electrical and magnetic fields, stress and strain fields, magnetization (through magnetic flux density) and polarization (through electric displacement) and von-Mises stress, besides the effective magneto-electro-mechanical properties.

The general homogenization method applied to multiferroics has no limitations regarding volume fraction or shape of the constituents involved and is based upon assumptions of periodicity of the microstructure and the separation of the microstructure scale through a proper asymptotic expansion[18]. The mathematical theory of homogenization accommodates the phase interaction in characterising both the macro- and micro-mechanical behaviours of the composite material. i.e., the method permits the introduction of different field equations in a microscopic scale to each constituent of a composite while following the representative volume element (RVE) notion. This study considers multiferroic materials that respond linearly to changes in the electric field, electric displacement, mechanical stress, strain as well as magnetic field. Let $\mathbf{\Omega}$ be a fixed domain in $\mathbf{x}$–space. We consider an auxiliary $\mathbf{y}$–space divided into parallelepiped periods $\mathbf{Y}$. For a linear anisotropic magnetoelectric material, generated through the periodic repetition of a *unit cell* representing the smallest sample of heterogeneity of the material domain $\mathbf{\Omega}$, the governng equations are given below;
force equilibrium equation,

$$\sigma_{ij,j} + b_i = \rho \ddot{u}_i \tag{S4}$$

strain-mechanical displacement relation,

$$\epsilon_{ij} = \frac{1}{2}(u_{i,j} + u_{j,i}), \tag{S5}$$

electrical (magnetic) field-electrical (magnetic) potential relations

$$\left.\begin{aligned} E_i &= -\varphi_{,i} \\ H_i &= -\psi_{,i} \end{aligned}\right\} \tag{S6}$$

and the quasistatic steady-state Maxwell's equations for electromagnetic phenomena,

$$\left.\begin{aligned} D_{i,i} &= 0 \\ B_{i,i} &= 0 \end{aligned}\right\} \tag{S7}$$

In a multiferroic solid, the mechanical, electrical and magnetic variables are related by constitutive relations. For small deformations, the linear constitutive laws of multiferroics in the absence of heat flux are given by

$$\sigma_{ij} = C^{EH}_{ijkl}\epsilon_{kl} - e_{kij}E_k - e^{M}_{kij}H_k \tag{S8}$$

$$D_i = e_{ijk}\epsilon_{jk} + \kappa^{\epsilon H}_{ij}E_j + \alpha_{ij}H_j \tag{S9}$$

$$B_i = e^{M}_{ijk}\epsilon_{jk} + \alpha_{ji}E_j + \mu^{\epsilon E}_{ij}H_j \tag{S10}$$

Here σ, ϵ, $\mathbf{D}$, $\mathbf{B}$ are stress, strain, displacement, body force, mass density, electric displacement vector, and magnetic flux density respectively. $\mathbf{C^{EH}}$, $\mathbf{e}$, $\mathbf{e^M}$, $\kappa^{\epsilon\mathbf{H}}$ and $\mu^{\epsilon\mathbf{E}}$ are stiffness, strain to (electric, magnetic) field coupling constants (or piezo-electric and -magnetic coefficients), permittivity (dielectric) and (magnetic) permeability respectively. Considering the standard homogenization procedure, the material functions $\mathbf{C^{EH}}$, $\mathbf{e}$, $\mathbf{e^M}$, $\kappa^{\epsilon\mathbf{H}}$ and $\mu^{\epsilon\mathbf{E}}$, are considered to be $\mathbf{Y}$-periodic functions in the unit cell

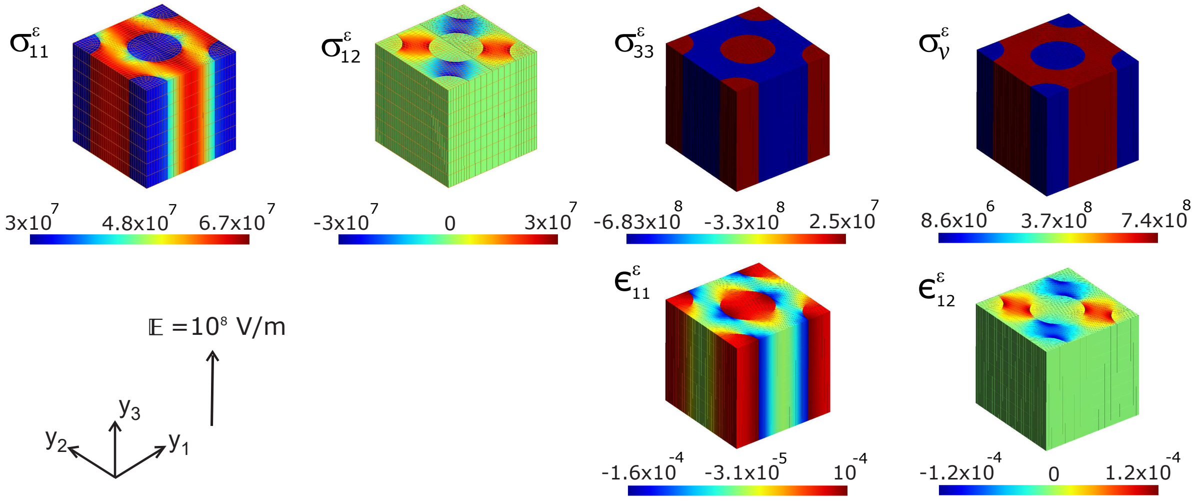

Figure S4. Map of local fields (computed at the nodal points of the FEM) of magnetoelectric composite $BaTiO_3$–$CoFe_2O_4$, *viz.*, the stress $\sigma^{\varepsilon}_{ij}$ (N/m^2) , strain $\epsilon^{\varepsilon}_{ij}$, and the equivalent von Mises stress σ^{ε}_{v} (N/m^2), upon applying electric field $\mathbb{E}$ of $10^8 V/m$ on the honeycomb unit cell. Here the magnetic $CoFe_2O_4$ cylindrical pillars are surrounded by ferroelectric $BaTiO_3$ matrix and both are aligned towards the y_3 axis of the local coordinate system. Images drawn using Inkscape 0.92.4 (https://www.inkscape.org) under GNU General Public License and Gmsh version 2.15.0 (http://gmsh.info/) under GNU General Public License.

domain defined as $\mathbf{Y} = [0, Y_1] \times [0, Y_2] \times [0, Y_3]$[18]. Here the two scales $\mathbf{x}$ and $\mathbf{y}$ are spatial variables where $\mathbf{x}$ is a macroscopic quantity and $\mathbf{y}$ is a microscopic one[19]. The functions involved in this expansion are assumed to be dependent on these two variables, where one (i.e., $\mathbf{x}$) describing the "global" or average response of the structure and the other (i.e., $\mathbf{y}$) describing the "local" or microstructural behaviour. Here the two variables $\mathbf{x}$ and $\mathbf{x}/\varepsilon$ take into account the two scales of the homogenization; the $\mathbf{x}$ variable is the macroscopic variable, whereas the $\mathbf{x}/\varepsilon$ variable takes into account the *microscopic* geometry.

The energy functional of the system consists of contributions from magnetic and electric parts and the magnetoelectric coupling component, which describe the effective interaction between order parameters of each phase. Let the surface Γ of a magneto-electro-elastic body $\boldsymbol{\Omega}$ be subjected to prescribed surface traction $\overline{t_k}$ and surface charge per unit area $\overline{\sigma}$ and normal magnetic flux density $\overline{B_n}$. The energy functional[20] for a magnetoelectric multiferroic can be written as,

$$\begin{aligned} G(\mathbf{u}^{\varepsilon}, \varphi^{\varepsilon}, \psi^{\varepsilon}) = \int_{\varepsilon} \Big[\frac{1}{2} C^{EH\ \varepsilon}_{ijkl}(\mathbf{x}, \mathbf{y}) \epsilon^{\varepsilon}_{ij} \epsilon^{\varepsilon}_{kl} \\ - e^{\varepsilon}_{ikl}(\mathbf{x}, \mathbf{y}) E^{\varepsilon}_{i} \epsilon^{\varepsilon}_{kl} - e^{M\ \varepsilon}_{ikl}(\mathbf{x}, \mathbf{y}) H^{\varepsilon}_{i} \epsilon^{\varepsilon}_{kl} \\ - \frac{1}{2} \kappa^{\epsilon H\ \varepsilon}_{ij}(\mathbf{x}, \mathbf{y}) E^{\varepsilon}_{i} E^{\varepsilon}_{j} - \alpha^{\varepsilon}_{ij}(\mathbf{x}, \mathbf{y}) E^{\varepsilon}_{i} H^{\varepsilon}_{j} \\ - \frac{1}{2} \mu^{\epsilon E\ \varepsilon}_{ij}(\mathbf{x}, \mathbf{y}) H^{\varepsilon}_{i} H^{\varepsilon}_{j} \Big] \mathrm{d}\varepsilon - \int_{\Gamma} \overline{t_k} u^{\varepsilon}_{k} \mathrm{d}\Gamma \\ + \int_{\Gamma} \overline{\sigma} \varphi^{\varepsilon} \mathrm{d}\Gamma + \int_{\Gamma} \overline{B_n} \psi^{\varepsilon} \mathrm{d}\Gamma \\ - \int_{\varepsilon} \frac{1}{2} \rho^{\varepsilon}(\mathbf{x}, \mathbf{y}) (\dot{u}^{\varepsilon}_{k})^2 \mathrm{d}\varepsilon - \int_{\varepsilon} b^{\varepsilon}_{k}(\mathbf{x}, \mathbf{y}) u^{\varepsilon}_{k} \mathrm{d}\varepsilon \quad \text{(S11)} \end{aligned}$$

Here $\rho^{\varepsilon}(\equiv \rho(\mathbf{x}, \mathbf{y}))$ is the density and $\mathbf{b}(\equiv \mathbf{b}(\mathbf{x}, \mathbf{y}))$ is the body force. The surface traction $\overline{t_k}$, surface charge density $\overline{\sigma}$ and normal magnetic flux density $\overline{B_n}$ are treated to be independent of the spatial scale ε. The spatial derivatives of displacement and potentials are taken and substituted into the Eqs. (S5) and (S6) and will eventually be substituted in the energy functional G. Applying calculus of variations, passing through the limit $\varepsilon \to 0$ and advance using asymptotic analysis one obtains the effective magneto-electro-elastic moduli, *viz.,* the homogenized elastic stiffnesses $\widetilde{C}^{EH}_{ijkl}$, piezoelectric coefficients $\widetilde{e}_{ijk}$, piezomagnetic coefficients $\widetilde{e}^{M}_{ijk}$, dielectric permittivities $\widetilde{\kappa}^{\epsilon H}_{ij}$, magnetic permeabilities $\widetilde{\mu}^{\epsilon E}_{ij}$

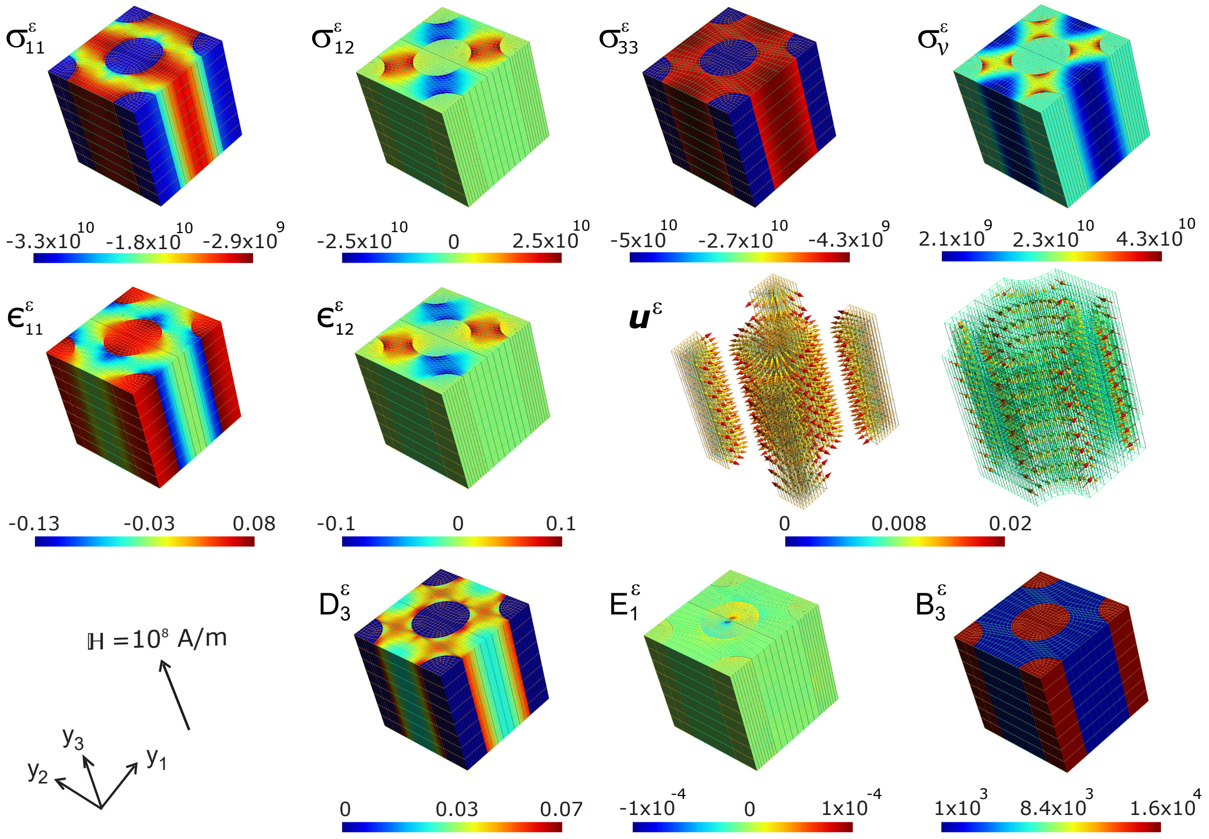

Figure S5. Map of local fields (computed at the nodal points of the FEM) of magnetoelectric composite $BaTiO_3$–$CoFe_2O_4$, *viz.*, the stress $\sigma^{\varepsilon}_{ij}$ (N/m^2) , strain $\epsilon^{\varepsilon}_{ij}$, displacement $\mathbf{u}^{\varepsilon}$ (m), the equivalent von Mises stress σ^{ε}_{v} (N/m^2), electric field E^{ε}_{j} (V/m), electric displacement $D^{\varepsilon}_{3}(C/m^2)$, and magnetic flux B^{ε}_{3} (Wb/m^2) upon applying electric field $\mathbb{E}$ of $10^8 V/m$ on the honeycomb unit cell. Here the magnetic $CoFe_2O_4$ cylindrical pillars are surrounded by ferroelectric $BaTiO_3$ matrix and both are aligned towards the y_3 axis of the local coordinate system. Images drawn using Inkscape 0.92.4 (https://www.inkscape.org) under GNU General Public License and Gmsh version 2.15.0 (http://gmsh.info/) under GNU General Public License.

and the magnetoelectric coupling coefficients $\widetilde{\alpha}_{ij}$ of the magnetoelectric multiferroic. In other words one could get a good approximation of the macroscopic behaviour of such a heterogeneous material by letting the parameter ε, which describes the fineness of the microscopic structure, tend to zero ($\varepsilon \to 0$) in the equations describing phenomena. And for a homogeneous material the physical properties does not depend on $\mathbf{x}$. The detailed theoretical analysis is given elsewhere[18]. (In all the expressions of this paper, it may be noticed to discern the difference in notations of asymptotic scale factor ε and the mechanical strain ϵ. All the microscopic quantities will carry the notation of ε to identify their local character). The local strain $\epsilon^{\varepsilon}_{ij}(\mathbf{x})$, electric field $E^{\varepsilon}_{j}(\mathbf{x})$ and the magnetic field $H^{\varepsilon}_{j}(\mathbf{x})$ too can be obtained in a similar way once the homogenized macroscopic problem is solved.

$$\epsilon^{\varepsilon}_{ij}(\mathbf{x}) = \epsilon^{0}_{ij}(\mathbf{x}) + \frac{\partial \eta^{mn}(\mathbf{x},\mathbf{y})}{\partial y_j} \times \epsilon_{mn}(u^0(\mathbf{x})) + \frac{\partial R^m(\mathbf{x},\mathbf{y})}{\partial y_j}\frac{\partial \varphi^0(\mathbf{x})}{\partial x_m} + \frac{\partial \Psi^m(\mathbf{x},\mathbf{y})}{\partial y_j}\frac{\partial \psi^0(\mathbf{x})}{\partial x_m} \quad \text{(S12)}$$

$$E^{\varepsilon}_{j}(\mathbf{x}) = -\Big[\frac{\partial \varphi^0(\mathbf{x})}{\partial x_j} + \frac{\partial \eta^{mn}(\mathbf{x},\mathbf{y})}{\partial y_j} \times \epsilon_{mn}(u^0(\mathbf{x})) + \frac{\partial R^m(\mathbf{x},\mathbf{y})}{\partial y_j}\frac{\partial \varphi^0(\mathbf{x})}{\partial x_m} + \frac{\partial \Psi^m(\mathbf{x},\mathbf{y})}{\partial y_j}\frac{\partial \psi^0(\mathbf{x})}{\partial x_m}\Big] \quad \text{(S13)}$$

$$H_j^{\varepsilon}(\mathbf{x}) = -\Big[\frac{\partial \psi^0(\mathbf{x})}{\partial x_j} + \frac{\partial \lambda^{mn}(\mathbf{x},\mathbf{y})}{\partial y_j} \times \epsilon_{mn}(u^0(\mathbf{x})) + \frac{\partial \Theta^m(\mathbf{x},\mathbf{y})}{\partial y_j}\frac{\partial \varphi^0(\mathbf{x})}{\partial x_m} + \frac{\partial Q^m(\mathbf{x},\mathbf{y})}{\partial y_j}\frac{\partial \psi^0(\mathbf{x})}{\partial x_m}\Big] \quad \text{(S14)}$$

The microscopic stress $\sigma_{ij}^{\varepsilon}(\mathbf{x})$, electrical displacement $D_i^{\varepsilon}(\mathbf{x})$ and magnetic flux densities $B_i^{\varepsilon}(\mathbf{x})$ at each point of the domain can be computed using the constitutive equations (S8)–(S10), and the field equations (S5) and (S6) as

$$\sigma_{ij}^{\varepsilon}(\mathbf{x}) = C_{ijkl}^{\varepsilon}(\mathbf{x})\Big(\frac{\partial u_k^0(\mathbf{x})}{\partial x_l} + \frac{\partial u_k^1(\mathbf{x},\mathbf{y})}{\partial y_l}\Big) - e_{kij}^{\varepsilon}(\mathbf{x})\Big(-\frac{\partial \varphi^0(\mathbf{x})}{\partial x_k} - \frac{\partial \varphi^1(\mathbf{x},\mathbf{y})}{\partial y_k}\Big) - e_{kij}^{M\ \varepsilon}(\mathbf{x})\Big(-\frac{\partial \psi^0(\mathbf{x})}{\partial x_k} - \frac{\partial \psi^1(\mathbf{x},\mathbf{y})}{\partial y_k}\Big) \quad \text{(S15)}$$

$$D_i^{\varepsilon}(\mathbf{x}) = e_{ijk}^{\varepsilon}(\mathbf{x})\Big(\frac{\partial u_j^0(\mathbf{x})}{\partial x_k} + \frac{\partial u_j^1(\mathbf{x},\mathbf{y})}{\partial y_k}\Big) + \kappa_{ij}^{\epsilon H\ \varepsilon}(\mathbf{x})\Big(-\frac{\partial \varphi^0(\mathbf{x})}{\partial x_j} - \frac{\partial \varphi^1(\mathbf{x},\mathbf{y})}{\partial y_j}\Big) + \alpha_{ij}^{\varepsilon}(\mathbf{x})\Big(-\frac{\partial \psi^0(\mathbf{x})}{\partial x_j} - \frac{\partial \psi^1(\mathbf{x},\mathbf{y})}{\partial y_j}\Big) \quad \text{(S16)}$$

$$B_i^{\varepsilon}(\mathbf{x}) = e_{ijk}^{M\ \varepsilon}(\mathbf{x})\Big(\frac{\partial u_j^0(\mathbf{x})}{\partial x_k} + \frac{\partial u_j^1(\mathbf{x},\mathbf{y})}{\partial y_k}\Big) + \alpha_{ji}^{\varepsilon}(\mathbf{x})\Big(-\frac{\partial \varphi^0(\mathbf{x})}{\partial x_j} - \frac{\partial \varphi^1(\mathbf{x},\mathbf{y})}{\partial y_j}\Big) + \mu_{ij}^{\epsilon E\ \varepsilon}(\mathbf{x})\Big(-\frac{\partial \psi^0(\mathbf{x})}{\partial x_j} - \frac{\partial \psi^1(\mathbf{x},\mathbf{y})}{\partial y_j}\Big) \quad \text{(S17)}$$

The flux represents the average normal component of the field times the surface area and in here the same definition is applicable about magnetic flux density **B**. The electrical displacement **D** would be essentially a measure of polarization **P** consequent to an applied electric field **E** in a general dielectric material. The equivalence of the average stress and homogenised stress can easily be expressed by applying the average operator $\langle . \rangle$ denoting $(\frac{1}{|Y|}\int_Y .dY)$, where Y is the domain size. Hence the average fields are computed to be

$$\langle \sigma_{ij} \rangle = \widetilde{C}_{ijkl}^{EH}\Big(\frac{\partial u_k^0(\mathbf{x})}{\partial x_l}\Big) + \widetilde{e}_{kij}\Big(\frac{\partial \varphi^0(\mathbf{x})}{\partial x_k}\Big) + \widetilde{e}_{kij}^{M}\Big(\frac{\partial \psi^0(\mathbf{x})}{\partial x_k}\Big) \quad \text{(S18)}$$

$$\langle D_i \rangle = \widetilde{e}_{ijk}\Big(\frac{\partial u_j^0(\mathbf{x})}{\partial x_k}\Big) - \widetilde{\kappa}_{ij}^{\epsilon H}\Big(\frac{\partial \varphi^0(\mathbf{x})}{\partial x_j}\Big) - \widetilde{\alpha}_{ij}\Big(\frac{\partial \psi^0(\mathbf{x})}{\partial x_j}\Big) \quad \text{(S19)}$$

$$\langle B_i \rangle = \widetilde{e}_{ijk}^{M}\Big(\frac{\partial u_j^0(\mathbf{x})}{\partial x_k}\Big) - \widetilde{\alpha}_{ji}\Big(\frac{\partial \varphi^0(\mathbf{x})}{\partial x_j}\Big) - \widetilde{\mu}_{ij}^{\epsilon E}\Big(\frac{\partial \psi^0(\mathbf{x})}{\partial x_j}\Big) \quad \text{(S20)}$$

These *macroscopic equations* (i.e. they do not contain y) can be computed once the homogenized solution for $\mathbf{u}^0$, φ^0 and ψ^0 and that of the corresponding fields *viz.,* $\frac{\partial u_j^0(\mathbf{x})}{\partial x_k}$, $\frac{\partial \varphi^0(\mathbf{x})}{\partial x_j}$ and $\frac{\partial \psi^0(\mathbf{x})}{\partial x_j}$ are prescribed. This *postulate* is equally applicable for the case with the microscopic fields depicted in equations Eqs. (2)–(4) from the main text and Eqs. (S12)–(S14) for the microscopic stress, electrical displacement and flux densities given in equations (S15)–(S17).

III. NUMERICAL IMPLEMENTATION

In order to solve the microscopic system of equations resulting form homogenization we developed a finite element (FEM) formulation and the details are given elsewhere[18]. A three-dimensional (3D) multiferroic finite element is conceived with five degrees of freedom (DOF)-three DOFs for spatial displacements and one each for electric and magnetic potentials. Eight-noded isoparametric elements with $2 \times 2 \times 2$ Gauss-point integration are used obtain solutions. Altogether there were nine microscopic equations that should be solved for as much number of unknowns namely the characteristic functions $\chi_i^{mn}, \eta^{mn}, \lambda^{mn}, R^m, \Phi_i^m, \Theta^m, Q^m, \Psi^m$ and Γ_k^m, where the indices m, n = 1, 2, 3[18]. The problem is reduced to standard variational FEM, after the usual approximations of finite element formulation and can be expressed concisely as

$$\boldsymbol{K}\boldsymbol{u} = \boldsymbol{f} \quad \text{(S21)}$$

where $\boldsymbol{K}$ is the global stiffness matrix, $\boldsymbol{u}$ is the vector of unknown functions and $\boldsymbol{f}$ is the load vector.

Magnetoelectric multiferroic material, in general,

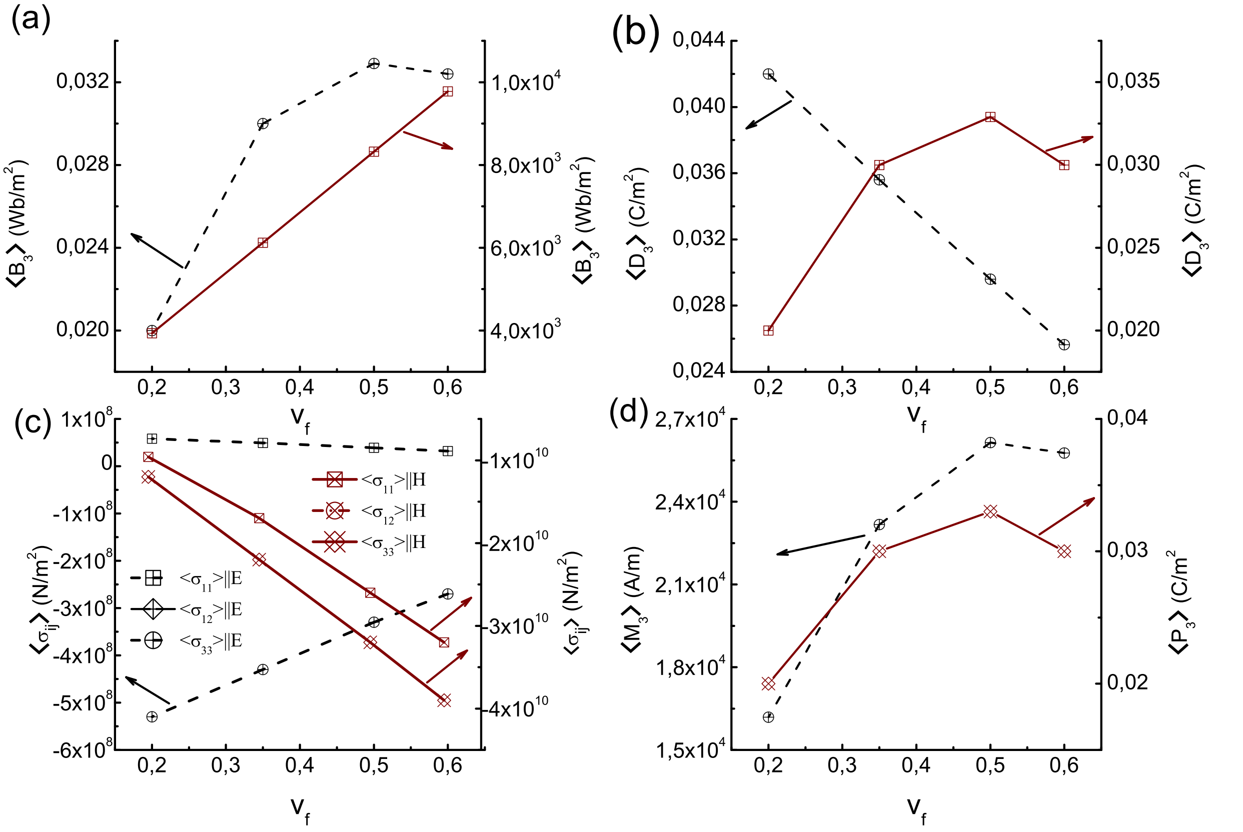

Figure S6. Plots of averages computed for a honeycomb lattice; (a) magnetic flux $\langle \mathrm{B}_3 \rangle$ (Wb/m^2), (b) electric displacement $\langle \mathrm{D}_3 \rangle$ (C/m^2), (c) stress $\langle \sigma_{ij} \rangle$ (N/m^2), (d) the magnetization $\langle \mathrm{M}_3 \rangle$ (A/m) and polarization $\langle \mathrm{P}_3 \rangle$ (C/m^2) upon applying a biasing electric field ($\mathbb{E}$) (dashed line plots) and magnetic field ($\mathbb{H}$) (line plots) separately on magnetoelectric composite of $BaTiO_3$–$CoFe_2O_4$ at various volume fractions (v_f) of the FM fibrous material $CoFe_2O_4$ of the composite.

can be considered as an aggregate of single crystalline crystallites/grains and hence the system altogether would be polycrystalline nature. The unit cell or the representative volume element (RVE) conceived in this work is a volume containing a sufficiently large number of crystallites or grains that its properties can be considered as equivalent to that of the macroscopic sample. The electric polarization **P** as well as the magnetization **M** interspersed inside the crystallites could be mapped using using some coordinate system. In a sense the underlying crystal orientation can encompass the orientations of **P** or **M**. Thus we introduce the Euler angles (φ, θ, ψ) to quantify the crystal orientations of a multiferroic polycrystal (Fig. S1), as the crystallites in an as-grown sample are randomly oriented in the lattice space and hence require three angles to describe its orientation with reference to a fixed coordinate system. Here we use the so called *x-convention*, where the first and third rotation is through the y-axis (here it is y_2'–axis)and the second rotation is through the intermediate x-axis (here it is y_1'–axis). Thus all the physical quantities $\lambda'_{ijklmn\ldots}(\mathbf{y}')$ expressed in a crystallographic coordinate system $\mathbf{y}'$ would be coordinate-transformed to the local coordinate system $\mathbf{y}$ according to the following scheme

$$\lambda_{ijkl\ldots}(\mathbf{y}) = e_{im}e_{jn}e_{kp}e_{lq}\ldots\widetilde{\lambda'}_{mnpq\ldots}(\mathbf{y}') \quad \text{(S22)}$$

before it is introduced for homogenization. (i.e., the FE and FM materials' electromechanical prop-

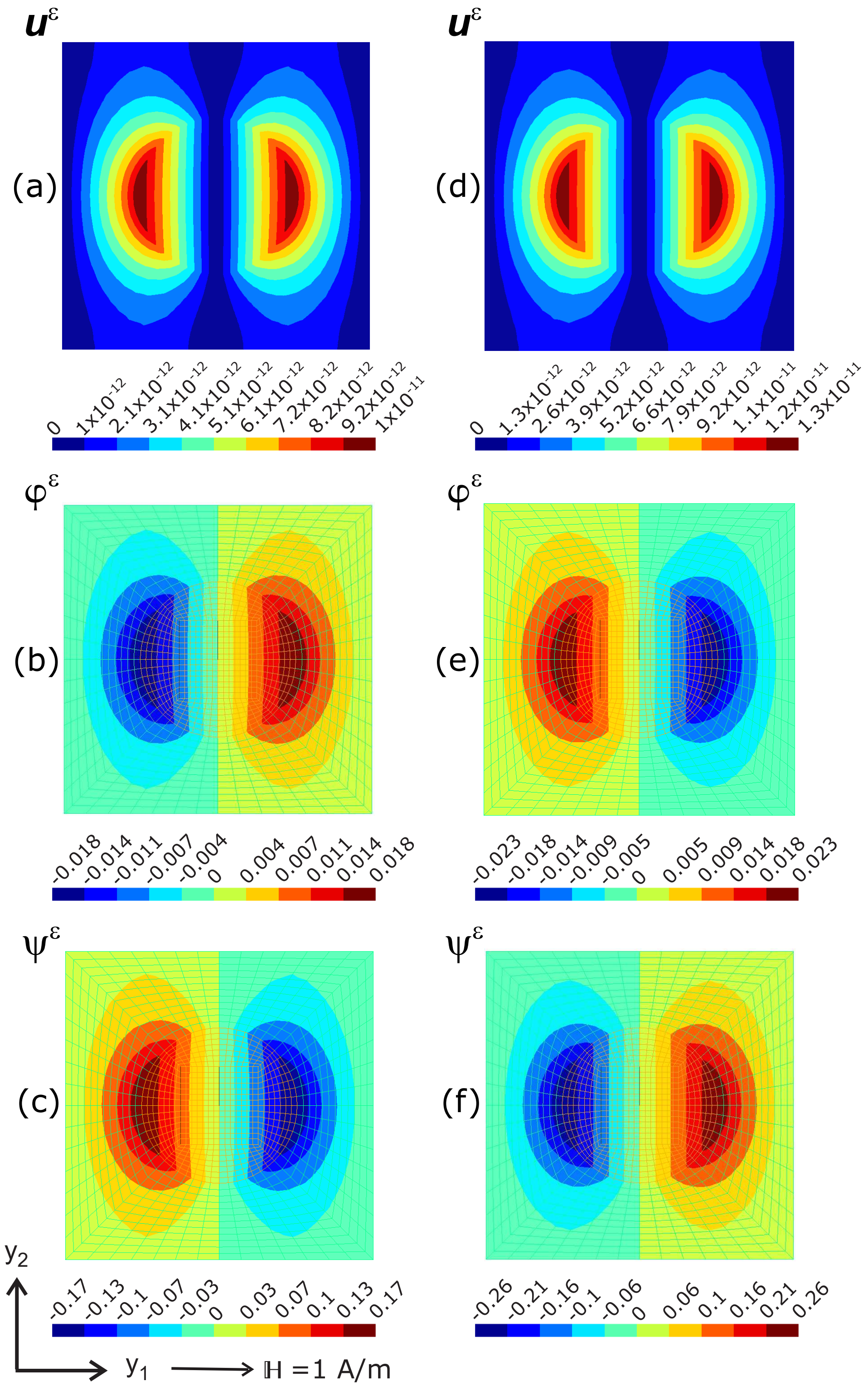

Figure S7. Plots of microscopic field profiles of displacement $\mathbf{u}^{\varepsilon}$ (m), electric potential φ^{ε} (V) and magnetic scalar potential ψ^{ε} (A), upon applying in–plane magnetic field $\mathbb{H}_x = 1\ A/m$ along the x–axis on magnetoelectric composite of $BaTiO_3$–$CoFe_2O_4$ at volume fraction ($v_f = 0.2$) of the fibre. (a–c) BTO fibres embedded in CFO matrix. (d–f) CFO fibres embedded in BTO matrix. Images drawn using Inkscape 0.92.4 (https://www.inkscape.org) under GNU General Public License and Gmsh version 2.15.0 (http://gmsh.info/) under GNU General Public License.

erty data entered into the homogenization program are obtained with respect to the crystallographic coordinates.) Here $e_{\mu\nu}$ are the Euler transformation matrices[21].

In the FEM simulation of the homogenization, a multiferroic crystallite is represented by a finite element in the unit cell. Thus we have a polycrystalline unite cell having as much number of crystallites as the number of finite elements by which it is discretized. As-grown FE (or for that matter FM) polycrystal, often ends up in a near complete compensation of polarization (or magnetization) and the material consequently exhibit very small, if any, electric (or magnetic) effect until they are poled by the application of an electric (magnetic) field. The orientation distribution of the crystallites (grains) in such a polycrystalline material would be uniform with a standard deviation $\sigma \to \infty$ before poling (application of electric/magnetic field) and that after poling would best be represented by a distribution function with $\sigma \to 0$. Thus, any pragmatic configuration of orientation distribution of grains in multiferroic material would fit in a Gaussian distribution defined by the probability distribution function

$$f(\alpha \mid \mu, \sigma) = \frac{1}{(\sigma\sqrt{2\pi})} \exp - \left[\frac{(\alpha - \mu)^2}{2\sigma^2} \right] \quad \text{(S23)}$$

where μ and σ are the the mean and the standard deviation of the angles α (which stands for the Euler angles $(\phi, \theta, \vartheta)$). The convergence of magnetoelectric properties with unit cell size allows us to determine the simulation-space independent, equivalent magnetoelectric properties of the composite. Convergence analyses, on magnetoelectric composites reveal that accuracy one derives from descretizing the unit cell (in other words sampling of the unit cells with more number of grains or less number) is minimal above 1000 elements (grains)[22]. Consequently, we kept unit cells' sizes greater than 1000 finite elements in this study.

IV. RESULTS

The elemental averages of local fields computed from the nodal values for each element of the FEM of the unit cell are shown in Fig. S2. It would be interesting to see the strain curves of this analysis as it is shown in Fig. S3. The strains are a consequence to the response of the material to stress and here it is caused by the external electric field $\mathbb{E}$. The anomaly at the boundary between the phases is obvious here as was seen in the stress analysis. The tensile component along the y_1–axis ($\epsilon^{\varepsilon}_{11}$) acquires positive value in the FM fibre phase of the composite while it is seen oscillating from tensile to compressive strain in the FE matrix.

The local and global fields using a honeycomb lattice unit cell of the ME composite is studied to verify the robustness of the POSTMAT developed in this work. The constitution of the composite material is not changed and the homogenization is run first to characterise the equivalent magneto-electro-elastic properties and the macroscopic strain, electric and magnetic potentials and their derivatives. We have kept the stoichiometry of the composite at $0.65BaTiO_3$–$0.35CoFe_2O_4$ first as in the above study. (Here the local distribution diagrams are shown slanted to enhance the visibility of the picture.) Here only the local distributions are drawn omitting symmetric and marginal ones. For instance, $\sigma^{\varepsilon}_{11} \equiv \sigma^{\varepsilon}_{22}$ and hence $\sigma^{\varepsilon}_{22}$ is not shown in the picture.

We have conducted a numerical experiment to simulate the magnetoelectric composite system described in Kuo and Bhattacharya[23]. To mimic their model we first take an ME composite with $BaTiO_3$ fibres embedded in $CoFe_2O_4$ matrix with fibre volume $v_f = 0.2$ where an external average magnetic field $H_x = 1\ A/m$ is applied to the composite along the in-plane x–direction. The resulting local displacement ($\mathbf{u}^{\varepsilon}$), electric potential (φ^{ε}) and magnetic potential (ψ^{ε}) contours are plotted and is shown in Fig. S7(a–c). The next column of subplots i.e., Fig. S7(d–f) pertains to the case where the composite is composed of $CoFe_2O_4$ fibres (with CFO $v_f = 0.2$) embedded in $BaTiO_3$ matrix. Here in both cases a transverse 2D section of the profiles at the mid-point of y_3–axis of the composite microstructure is given. The profiles are in good accord with the ones shown in Kuo and Bhattacharya[23] while admitting that we have used single crystal $BaTiO_3$ as matrix instead of the ceramic matrix used by them. It is noticed that their model treats only anti-plane shear with in-plane electromagnetic fields and contrasts with ours where we have admitted all the shear components and electromagnetic fields.

---

* kpjayachandran@gmail.com

[1] H. Schmid, Ferroelectrics **162**, 317 (1994).

[2] M. Fiebig, T. Lottermoser, D. Meier, and M. Trassin, Nat. Rev. Mater. **1**, 16046 (2016).
[3] N. A. Hill, J. Phys. Chem. B **104**, 6694 (2000).
[4] N. A. Spaldin and R. Ramesh, Nat Mater **18**, 203 (2019), ISSN 1476–4660, URL https://doi.org/10.1038/s41563-018-0275-2.
[5] M. Avellaneda and G. Harshe, J. Intell. Mater. Syst. Struct. **5**, 501 (1994).
[6] J. van den Boomgaard and R. A. J. Born, J. Mater. Sci. **13**, 1538 (1978), ISSN 1573-4803.
[7] R. E. Newnham, D. P. Skinner, and L. E. Cross, Mater. Res. Bull. **13**, 525 (1978), ISSN 0025-5408, URL http://www.sciencedirect.com/science/article/pii/0025540878901617.
[8] C. A. F. Vaz, J Phys-Condens Mat **24**, 333201 (2012), URL https://doi.org/10.1088/2F0953-8984/2F24/2F33/2F333201.
[9] J. Zhai, Z. Xing, S. Dong, J. Li, and D. Viehland, J Amer Ceram Soc **91**, 351 (2008), ISSN 1551-2916.
[10] S. Sahoo, S. Polisetty, C.-G. Duan, S. S. Jaswal, E. Y. Tsymbal, and C. Binek, Phys. Rev. B **76**, 092108 (2007), URL https://link.aps.org/doi/10.1103/PhysRevB.76.092108.
[11] Y.-H. Chu, L. W. Martin, M. B. Holcomb, M. Gajek, S.-J. Han, Q. He, N. Balke, C.-H. Yang, D. Lee, W. Hu, et al., Nat Mater **7**, 478 (2008), URL https://doi.org/10.1038/nmat2184.
[12] M. Endo, S. Kanai, S. Ikeda, F. Matsukura, and H. Ohno, Appl Phys Lett **96**, 212503 (2010), https://doi.org/10.1063/1.3429592, URL https://doi.org/10.1063/1.3429592.
[13] H. Lu, T. A. George, Y. Wang, I. Ketsman, J. D. Burton, C.-W. Bark, S. Ryu, D. J. Kim, J. Wang, C. Binek, et al., Appl Phys Lett **100**, 232904 (2012), https://doi.org/10.1063/1.4726427, URL https://doi.org/10.1063/1.4726427.
[14] Z. Chu, M. PourhosseiniAsl, and S. Dong, J. Phys. D: Appl. Phys. **51**, 243001 (2018), URL http://stacks.iop.org/0022-3727/51/i=24/a=243001.
[15] J. D. Burton and E. Y. Tsymbal, Phys. Rev. B **80**, 174406 (2009), URL https://link.aps.org/doi/10.1103/PhysRevB.80.174406.
[16] Y. Uetsuji, T. Wada, and K. Tsuchiya, Comput Mater Sci **158**, 159 (2019), ISSN 0927-0256, URL http://www.sciencedirect.com/science/article/pii/S0927025618307407.
[17] L. D. Landau and E. M. Lifshitz, *Electrodynamics of Continuous Media* (Pergamon, Oxford, 1960).
[18] K. P. Jayachandran, J. M. Guedes, and H. C. Rodrigues, J Intel Mat Syst Str **25**, 1243 (2014).
[19] E. Sanchez-Palencia, *Non-homogeneous media and vibration theory, Lecture notes in physics 127* (Springer-Verlag, Berlin, 1980).
[20] Q. H. Qin and Q. S. Yang, *Macro-Micro Theory on Multifield Coupling Behavior of Heterogeneous Materials* (Springer Berlin, Heidelberg, 2009).
[21] H. Goldstein, *Classical Mechanics* (Addison-Wesley, Reading, MA, 1978).
[22] K. P. Jayachandran, J. F. A. Madeira, J. M. Guedes, and H. C. Rodrigues, Comp Mater Sci **148**, 190 (2018), ISSN 0927–0256.
[23] H.-Y. Kuo and K. Bhattacharya, Mech Mater **60**, 159 (2013).
